# Recent Progress in Fabrication and Application of BN Nanostructures and BN-Based Nanohybrids

**DOI:** 10.3390/nano12162810

**Published:** 2022-08-16

**Authors:** Dmitry V. Shtansky, Andrei T. Matveev, Elizaveta S. Permyakova, Denis V. Leybo, Anton S. Konopatsky, Pavel B. Sorokin

**Affiliations:** Labotoary of Inorganic Nanomaterials, National University of Science and Technology “MISiS”, Leninsky Prospect 4, 119049 Moscow, Russia

**Keywords:** hexagonal BN, nanostructures, nanohybrids, fabrication, application

## Abstract

Due to its unique physical, chemical, and mechanical properties, such as a low specific density, large specific surface area, excellent thermal stability, oxidation resistance, low friction, good dispersion stability, enhanced adsorbing capacity, large interlayer shear force, and wide bandgap, hexagonal boron nitride (*h*-BN) nanostructures are of great interest in many fields. These include, but are not limited to, (i) heterogeneous catalysts, (ii) promising nanocarriers for targeted drug delivery to tumor cells and nanoparticles containing therapeutic agents to fight bacterial and fungal infections, (iii) reinforcing phases in metal, ceramics, and polymer matrix composites, (iv) additives to liquid lubricants, (v) substrates for surface enhanced Raman spectroscopy, (vi) agents for boron neutron capture therapy, (vii) water purifiers, (viii) gas and biological sensors, and (ix) quantum dots, single photon emitters, and heterostructures for electronic, plasmonic, optical, optoelectronic, semiconductor, and magnetic devices. All of these areas are developing rapidly. Thus, the goal of this review is to analyze the critical mass of knowledge and the current state-of-the-art in the field of BN-based nanomaterial fabrication and application based on their amazing properties.

## 1. Introduction

**Figure 1:**
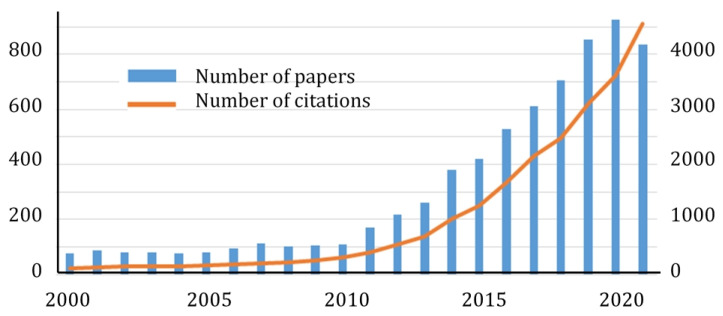
Statistics of publications and citations when searching for keywords “BN nanostructures” in the Web of Science database.

In recent years, the number of works devoted to hexagonal boron nitride (*h*-BN) nanostructures has grown rapidly, and the scope of their application has expanded significantly. Statistics of publications and citations when searching for keywords “BN nanostructures” in the Web of Science database are shown in Figure 1. The number of annual articles has already exceeded 900, and the number of citations is more than 4000. The main goal of this review is to provide a critical analysis of the state-of-the-art in the field of *h*-BN nanostructures based on a review of the most recent works in order to demonstrate their promise in many critical areas of modern science and technology.

The main structural blocks of the sp^2^-bonded BN are six-membered rings formed by B and N atoms. Regardless of the vertical stacking sequence, the interplanar spacing is ∼0.333 nm. In the simplest AA stacking, each B or N atom sits on top of its B or N counterpart from one layer to another (P$\bar{6m}$2 symmetry). In the case of *h*-BN with P63/mmc symmetry, every other layer is rotated by 180° around the [0001] axis and every B atom in the parallel hexagons sits on top of a N atom (A-A′ stacking). Rhombohedral BN (*r*-BN) has three-layer ABC stacking (R3m symmetry), in which, hexagons are displaced by a vector of *a*/3 in a <01$\bar{1}$0> direction, keeping the same interplanar spacing as *h*-BN. The sp^2^-bonded BN is often highly defective, which is a great advantage for some applications (e.g., catalysis, photocatalysis, water splitting and purification, sensor devices) and a strong disadvantage for others (typically optics and electronics). A mixture of *h*-BN and *r*-BN crystallites obeying specific orientation relationships [1] is often observed in the sp^2^-bonded materials. This leads to a local bending of BN atomic planes (this type of BN is called turbostratic BN (*t*-BN)). Edge dislocations are common within the *t*-BN. Inside the turbostratic structure, the interlayer distance is increased, which leads to a weakening of the optical and electronic interactions between adjacent layers. Less common is AB of symmetry (so-called Bernal stacking), in which, the centers of hexagons in one layer are vertically aligned with atoms in adjacent layers [2]. AB stacking was observed in chemically exfoliated [3] and synthesized [4] few-layer *h*-BN.

Hexagonal BN can be prepared as 0D (fullerenes, quantum dots (QDs)), 1D (nanotubes (NTs), nanorods, atomically flat grain boundaries), 2D (monolayer, nanosheets (NSs)), and 3D materials (films, nanoparticles (NPs)). In the 1D configuration, *h*-BN is of great interest for electronic devices as an atomically sharp AA′/AB stacking boundary [5]. Although graphite-like *h*-BN is the structural analog of graphene (Gr) and their physical and chemical properties are often compared, many properties, such as their color, electric conductivity, and oxidation resistance, are different. Hexagonal BN is a wide-band-gap (~6.0 eV) insulator [6]. The versatile use of *h*-BN nanostructures is associated with an interesting combination of properties: a low specific density, high specific surface area, high thermal conductivity and chemical stability, insulation, superior oxidation resistance, a natural optical hyperbolic behavior, and an unusually bright deep-UV emission. In contrast to Gr, which is brittle, single-crystal monolayer *h*-BN demonstrated high fracture toughness with an effective energy release rate of up to one order of magnitude higher than that predicted by Griffith low and reported for Gr [7]. Recent results have shown that atomically thin *h*-BN exhibits wetting translucency similar to that of Gr [8]. The piezoelectric characteristics were theoretically predicted in the monolayer *h*-BN, which has no center of symmetry [9], and subsequently confirmed experimentally in the *h*-BN monolayer [10] and defective *h*-BN NTs [11].

Hexagonal BN nanostructures are widely used as additive, thin film, support, encapsulant, carrier, insulator, and barrier material. For nanophotonic, optoelectronic, and sensing applications, it is also considered as a host material for active atomic vacancies/defects. Hexagonal BN is commonly used as a low-loss “dielectric spacer” in many photoelectronic devices.

**Figure 2:**
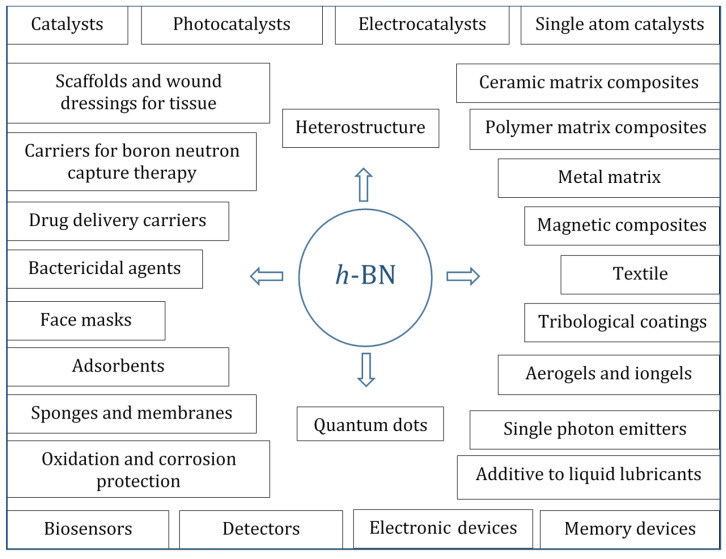
Applications of *h*-BN nanostructures.

Currently, *h*-BN nanomaterials are used in many areas, such as catalysis, photocatalysis, biomedicine (agent for antibacterial, antifungal, antitumor, and boron neutron capture therapy), environmental industry (biodetectors, membranes, and filters), tribology (additive to solid and liquid lubricants), energy (components of cathodes and capacitors in batteries), nanoelectronics, quantum optics and photonics, and deep UV optoelectronics (Figure 2). They are also widely utilized as a reinforcing and/or heat transfer phase in metal, ceramic, and polymer matrix composites, as a modifier of textile materials and soft magnetic composites, and as a filler for heat-insulating aerogels and ionogels. Many of the unique properties of *h*-BN are associated with point defects, formed either as a result of material production or by subsequent mechanical, chemical, or irradiation treatments. Therefore, their precise controlling is of a great importance. Defect engineering by hydrogen plasma treatment and high-temperature annealing was shown to be an effective tool to control point vacancies and oxygen-related defects [12]. However, the identification of point defects in *h*-BN is still a challenge. Recently, significant progress has been made in studying the distribution of electric fields at the atomic level using advanced differential phase contrast imaging. Using this method, it is possible to map and measure enhanced electric fields around B monovacancies with respect to an ideal lattice [13].

## 2. Fabrication and Surface Functionalization

BN flakes with sp^2^-hybridization were obtained as far back as in 1842 [14]. Hexagonal BNNTs were first synthesized in 1995 using an arc-discharge method [15]. However, the wide application of BNNTs is limited by the low material purity and the inability to control the aspect ratio. A successful step towards obtaining well-dispersed individualized BNNTs is the use of conjugated polymers capable of sorting BNNTs into individual species [16]. In 2016, oxygen containing *h*-BN nanostructures of various morphologies having hollow and solid cores and smooth and petalled surfaces (with numerous thin-walled *h*-BN petals) were synthesized using a boron-oxide-assisted chemical vapor deposition (BO-CVD) process [17]. BN nanostructures are obtained by bottom-up and top-down approaches. Several aspects of BN synthesis have been highlighted in recent reviews, mostly in relation to specific applications [18,19,20,21,22,23,24,25].

### 2.1. Bottom-Up Approach

Disc-shaped *h*-BNNPs approximately 20 nm in size were synthesized by the microwave heating of a mixture of boric acid and melamine [26]. The use of microwave energy as an alternative heating source made it possible to reduce the reaction time by a factor of three compared to conventional heating. The temperature range for the synthesis reaction was 1130–1210 °C, and the duration of the synthesis was 90 min.

**Figure 3:**
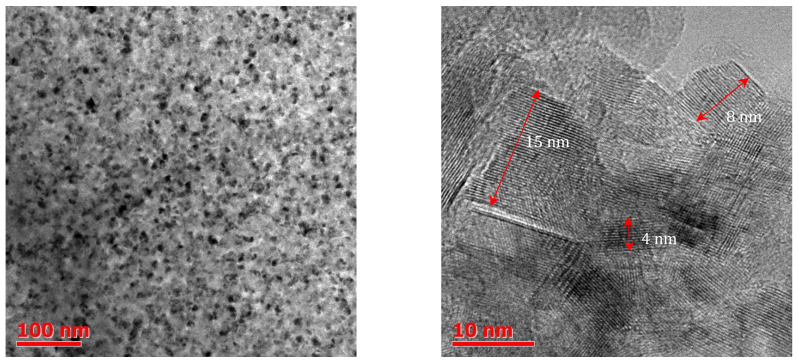
Homogeneous BNNSs with an average size of 10 nm synthesized by one-stage low-temperature ammonolysis of boric acid (ammonothermal dehydration method).

Boric acid and ammonia were used as boron and nitrogen sources in most *h*-BN synthesis protocols. The fundamental difference in the recently proposed new method is the treatment of ammonia gas with boric acid at room temperature [27]. During the reaction, part of the hydroxyl groups is replaced by amino groups, which leads to the formation of an ammonium borate hydrate compound (NH_4_)_2_B_4_O_7_ × 4H_2_O (ABH) containing B-N bonds already at room temperature. The further heating of ABH in a stream of ammonia causes subsequent ammonolysis and dehydration, so this method is called an ammonothermic dehydration process. Synthesis at 550 °C for 24 h made it possible to obtain *h*-BN nanocrystals approximately 10 nm (Figure 3) in size at a 10% yield. With an increase in the synthesis temperature to 1000 °C, the yield gradually increased. This method paves the way for a new cost-effective and scalable bottom-up approach for the fabrication of *h*-BN nanocrystals.

Multiwall BNNTs were obtained by CVD at a relatively low temperature of 1050 °C using colemanite as a B precursor and an Fe_2_O_3_ catalyst [28]. BN nanoribbons were prepared from BNNTs by applying high pressure [29]. The rupture of NTs led to the formation of new morphological types of *h*-BN, including nanoribbons encapsulated inside BNNTs. Wrinkled *h*-BN nanofoam was obtained from graphite by a simultaneous carbothermic reduction and nitriding of boron oxide powder [30]. Interestingly, in this approach, the graphite NSs acted as a carbon source for the reduction reaction and as a rough template for *h*-BN growth.

The highly crystalline *h*-BNNPs (FWHM E2g of 11.07 cm^−1^) with a well-defined nanosheet morphology and large crystal size (2.7–8.4 μm) were successfully synthesized using a polymer-derived ceramics process [31]. The addition of melting point reduction agents (BaF_2_ and Li_3_N) made it possible to obtain *h*-BN powder at a relatively low temperature (1200 °C) and atmospheric pressure. Hexagonal BNNSs with large lateral sizes (a few microns) were also obtained by salt-assisted synthesis [32].

Few-layer *h*-BN films with micron-sized grains were epitaxially grown by CVD on the surface of a Cu film at a temperature of 1100 °C [33]. The thermal CVD process was carried out in a low-pressure chamber (500 mbar) in a gaseous mixture of NH_3_ and B_2_H_6_ with the addition of H_2_ and Ar. According to TEM analysis, approximately 92% of *h*-BN films had one to three atomic layers.

High-quality multilayer *h*-BN with a controlled thickness (5 to 50 nm) was obtained by the vapor–liquid–solid method using a molten Fe_82_B_18_ alloy and N_2_ as reagents [34]. A thin Fe-B alloy plate was placed over the sapphire and annealed at a temperature above the melting point of the alloy (1250 °C) for 60 min in an Ar/H_2_ flow (300/50 cm^3^/min). Further treatment at this temperature was carried out in a N_2_ flow (300 cm^3^/min) for 60 min. Liquid Fe_82_B_18_ not only supplies boron, but also continuously dissociates nitrogen atoms, supporting the growth of *h*-BN. The 2D growth was strictly limited by the interface between the liquid Fe_82_B_18_ and the sapphire substrate. After cooling, the Fe-B alloy could be easily separated, leaving the *h*-BN multilayer on the sapphire substrate.

Large single-crystal *h*-BN with a transverse size of several millimeters is of interest for transistors and photoelectronic devices. Such transparent crystals were obtained by polycondensation, stabilization, and sintering with the addition of preceramic powder [35]. High-quality *h*-BN crystals were grown at an atmospheric pressure using pure iron as a flux [36]. The narrow vibration Raman E2g peak (7.6 cm^−1^) and strong phonon-assisted peaks in the photoluminescence spectra evidenced the high quality of the resulting nanomaterial.

The direct growth of high-quality *h*-BN on dielectric/insulating/transparent substrates is important for electronic and optoelectronic applications. To address this important issue, Bansal et al. [37] studied the growth of *h*-BN on a (0001) C-plane and (11$\bar{2}$0) A-plane of sapphire (α-Al_2_O_3_). The obtained results show that the A-plane is not modified during CVD and, therefore, is a suitable substrate orientation for the growth of *h*-BN with ABAB staking. Few-layer *h*-BN was deposited on the sapphire substrate using the ion beam sputtering of an *h*-BN target in a NH_3_ atmosphere at a relatively low temperature of 700 °C [38]. Granular nanocrystalline *h*-BN films were grown on silicon (100) and C-sapphire substrates by low-pressure CVD at 1100 °C using ammonia borane [39]. Hexagonal BN is often used as a substrate for in situ Gr growth to fabricate Gr/*h*-BN heterostructures for nanoelectronics. Highly ordered epitaxial *h*-BN was obtained on Gr using a migration-enhanced metalorganic vapor phase epitaxy process [40]. This subject was recently reviewed [41].

### 2.2. Top-Down Approach

For the BN production on a laboratory scale, physical, chemical, and mechanical methods are used. Mechanical exfoliation in various mediums is the widest group of methods used to obtain BNNSs and BNNPs. Since the interlayer bond force between hexagonal layers is relatively weak, these layers are easy to separate with an external force. The mechanical exfoliation methods include blending, microfluidization, ball milling, and sonication.

#### 2.2.1. Microfluidization

Atomically thin BNNSs with a high aspect ratio of ~1500 were fabricated by microfluidization in a water/ethanol (1:1) solution for 50 cycles at each pressure of 50, 75, 100, and 125 MPa [42]. The exfoliation process takes only 30 min, provides a high yield of 70–76%, and produces approximately 400 mg of low-defect BNNSs. The as-prepared BNNSs were utilized in polymer matrix composites with high thermal conductivity.

#### 2.2.2. Ball Milling

The submicron *h*-BN powder preliminary synthesized by the high-temperature solid-phase method was exfoliated in a ball mill with the addition of water in a reciprocating mode [43]. This approach demonstrated BNNSs with a thickness of approximately 4–10 nm, a uniform size distribution, and a high yield of 73%. Various saccharides, such as fructose, glucose, maltose, and lactose, were used to improve the efficiency of BN exfoliation during ball milling [44]. Among the studied saccharides, the highest yield of few-layered BNNSs (29.4%) was achieved using glucose. In this mechanochemical exfoliation method, saccharides enhance the applied force of ball milling to promote BN delamination and prevent the reattachment of the nanosheets through their surface functionalization.

When developing ball-milling methods, special attention is paid to studying the influence of the processing medium on the exfoliation process. For example, processing in viscous hydroxyethyl cellulose leads to the formation of BNNSs with a lateral size of 400 nm and a thickness of approximately 2 nm and inhibits NPs agglomeration [45]. Yusupov et al. [46] obtained a high yield of uniform and undeformed BNNSs with an average size of 300 × 600 nm^2^ and a thickness of approximately 20–50 nm by the ball milling of micron-size BN particles in ethylene glycol with a high density of 1.1 g/m^3^. Importantly, the milling balls move along the periphery of the chamber, in contact with the walls. This regime creates tangential forces acting on BN particles and leads to their exfoliation. Ball milling with the addition of 2-furoic acid made it possible to achieve a very high yield (~98%) of BNNSs with a thickness of ~2 nm and a lateral size of up to 2 μm [47]. Ultrathin (with average thickness of 3.5 nm) *h*-BNNSs noncovalently functionalized by ionic liquid were prepared using a liquid ball milling method [48].

#### 2.2.3. Sonication

Recently, the highly efficient exfoliation of *h*-BN in water upon doping with oxygen was reported [49]. Oxygen atoms were introduced into the *h*-BN structure via oxidative heat treatment. After annealing in air at 1000 °C for 30 min, oxygen-doped *h*-BN is easily separated by sonication in water. This method provides a 1255% increase in BN yield at an average nanosheet thickness of 1.3 nm compared to untreated *h*-BN. The facilitation of the separation process is associated with the appearance of hydroxyl groups during oxidation, which are capable of forming hydrogen bonds that affect the binding energy (BE) of the *h*-BN layers. Few- and single-layer *h*-BNNSs were prepared using high-intensity sonication in a pressure batch reactor [50].

A hydroxylation approach for exfoliating *h*-BN using a thermal-assisted hydrolysis method was described [51]. Bulk *h*-BN was first sonicated in water for 120 min, and then the resulting hydroxyl-functionalized *h*-BN powder was heat-treated at 400 °C for 2 h. Rapid heating weakened the lip–lip interaction between the *h*-BN layers due to water evaporation, and subsequent ultrasonic treatment in water for 2 h led to the formation of BN nanoflakes with an average transverse size of ~0.58 μm and a thickness of 3–8 nm. The efficiency of this method was 37%. Another promising approach to efficient *h*-BN exfoliation in an aqueous medium is associated with the use of tannic acid [52], whose molecules have catechol/pyrogallol groups, which ensure their absorption onto the *h*-BN surface via π−π stacking. This results in lower specific surface energy and weaker lip–lip interaction between the *h*-BN layers. This method makes it possible to obtain three-four-layer BNNSs with an exfoliation yield of 42.2%. The resulting acid-functionalized *h*-BNNSs showed good dispersion in both water and isopropanol.

The effect of various surfactants (cationic, anionic, and nonionic) on the yield and stability of *h*-BNNSs was systematically studied [53]. It has been established that the material yield does not depend on the surfactant, but the dispersion stability obtained with ionic surfactants is significantly higher than with nonionic surfactants or water. Dispersions in ionic surfactants such as sodium dodecyl sulfate and cetyltrimethylammonium chloride remained stable for at least 24 days, whereas dispersions in water and nonionic surfactant solutions precipitated over time. Various solvents, such as dimethylformamide (DMF), dimethyl sulfoxide (DMSO), and isopropyl alcohol (IPA), were tested as liquid media for the sonication exfoliation method [54]. The best result was achieved with sonication for 2 h in a mixed IPA solvent with deionized water (3:7). Under optimized conditions, *h*-BNNSs were obtained with an average size of ~2 μm, a thickness of ~1.2 nm, and a concentration of 0.6 mg/mL with a yield of 90%. It has been proposed that a Lewis acid–base interaction mechanism is responsible for the *h*-BN exfoliation due to electron-deficient boron atoms.

A highly efficient and scalable sonication-assisted liquid-phase exfoliation has been reported with a yield of 72.5% for few-layer and defect-free BNNSs [55]. An IPA solution without any surface-active additives was used. The effect of several parameters on the BNNSs yield, such as the ultrasonic frequency and power, sonication time, and IPA/water volume ration, has been systematically investigated. The highest exfoliation efficiency of approximately 10% per hour was achieved under the optimal condition: 30 kHz, 315 W, and IPA/water = 40/60. The optimal IPA volume fraction was in the range of 40–50%.

The solvent–solvent interaction is key in screening high-performance systems for the efficient ultrasonic liquid exfoliation of layered materials. For example, aqueous solutions of two isomers NPA (1-propanol) and IPA (2-propanol) showed markedly different exfoliation efficiencies [56]. The yield of BNNSs when using an IPA aqueous solution is more than two times higher than the yield obtained using an aqueous NPA solution, while the optimal concentrations of alcohols also differ and are 19 mol% (IPA) and 14 mol% (NPA). In water, IPA and NPA molecules form dynamic clusters that exist for 1 to 10 picoseconds, and the cluster size plays an important role in the exfoliation process. Too large clusters do not easily penetrate the interlayer gap between *h*-BN, and too small clusters do not provide a steric effect in the interlayer gap. Only clusters of the optimal size can prevent the restoration of the layered *h*-BN structure after the ultrasonic explosion force of a microbubble overcomes the van der Waals force of the attraction of the *h*-BN layers.

#### 2.2.4. Chemical, Hydrothermal, and Cryogenic Exfoliation

In addition to mechanical exfoliations, BN nanostructures are obtained by chemical, hydrothermal, and cryogenic exfoliation. A low-cost and energy-efficient wet chemical exfoliation method was recently proposed [57]. This method is a modification of the Hummers method that uses strong oxidizers such as H_2_SO_4_, KMnO_4_, and H_2_O_2_. Commercial *h*-BN powder was heated to 75 °C in a mixture of sulfuric acid, KMnO_4_, and H_2_O_2_ during 24 h. The resulting product was washed several times with distilled water using a centrifuge at 4200 rpm until the pH of the supernatant reached seven. Few-layer edge-functionalized *h*-BNNSs with an average thickness of 1.8 nm and a size of 486 ± 51 nm were produced with a yield of more than 83%. Hexagonal BNNSs were obtained by a hydrothermal exfoliation using various polar solvents [58]. A high yield (~55%) of thin BNNSs (1–3 nm) with a lateral size of tens of micrometers and a concentration of ~4.13 mg/mL was achieved with LiCl-assisted hydrothermal exfoliation in IPA.

Hexagonal BNNPs less than 10 nm in diameter were obtained by the cryogenic method [59]. To achieve this, BN nanopowder with an average particle size of 100 nm was soaked in liquid nitrogen for 1 h and then subjected to thermal shock by rapid dispersion in a water–isopropanol solution (1:1) at room temperature. The resulting solution was sonicated in an industrial ultrasonic bath for 4 h. After double centrifugation at 12,000 rpm for 30 min, BNNPs with a size of 0.7–3.0 nm were collected. However, the cryogenic effect in this method is not obvious, since the sonication of *h*-BN powder in an aqueous isopropanol solution also allows for obtaining NPs.

For completeness, some physical methods for obtaining BNNPs should also be mentioned. Hexagonal BNNPs with an average diameter of 120 nm and a thickness of approximately 1.7 nm were obtained by processing micron-sized *h*-BN powder in the nitrogen plasma of a high-voltage arc discharge [60]. Although the yield of nanoparticles and the mechanism of their formation have not been reported, it can be assumed that the exfoliation occurs due to the high temperature and/or energy transfer from charged particles to BN particles.

A new radiation-induced reduction–exfoliation method was proposed for the facile one-step fabrication of BNNSs decorated with Ni NPs [61]. The method is based on the intercalation of Ni^2+^ ions into *h*-BN interlayers in an IPA/water medium due to the acid–base Lewis effect between Ni^2+^ ions and N atoms in *h*-BN. Then, the dispersion of Ni^2+^ intercalated *h*-BN was gamma irradiated. The reduction of Ni^2+^ ions during gamma irradiation causes the growth of Ni NPs, which, due to the volume expansion effect, leads to *h*-BN exfoliation. The resulting Ni/BN nanomaterials consisted of BNNSs less than 5 nm thick and 200 nm in size that were decorated with Ni NPs.

### 2.3. Surface Functionalization

Many *h*-BN applications require surface functionalization. This can be carried out by chemical treatment in salt and alkali solutions, heat treatment, or irradiation. The treatment of *h*-BN in thionyl chloride was shown to initiate corrosion and lead to the formation of hydroxyl and amino groups [62]. For the hydroxylation of the *h*-BN surface, as well as for the removal of boric acid formed during the temperature-activated *h*-BN decomposition, various types of heat treatment were proposed [63]. Recently, various synthetic approaches toward the functionalization of BN nanomaterials with OH groups have been discussed [64]. Stable *h*-BN aqueous dispersions were obtained using a combination of sonication and treatment with O_3_ and hydrogen peroxide [65].

## 3. Catalysts

### 3.1. Heterogeneous and Homogeneous Catalysts

**Figure 4:**
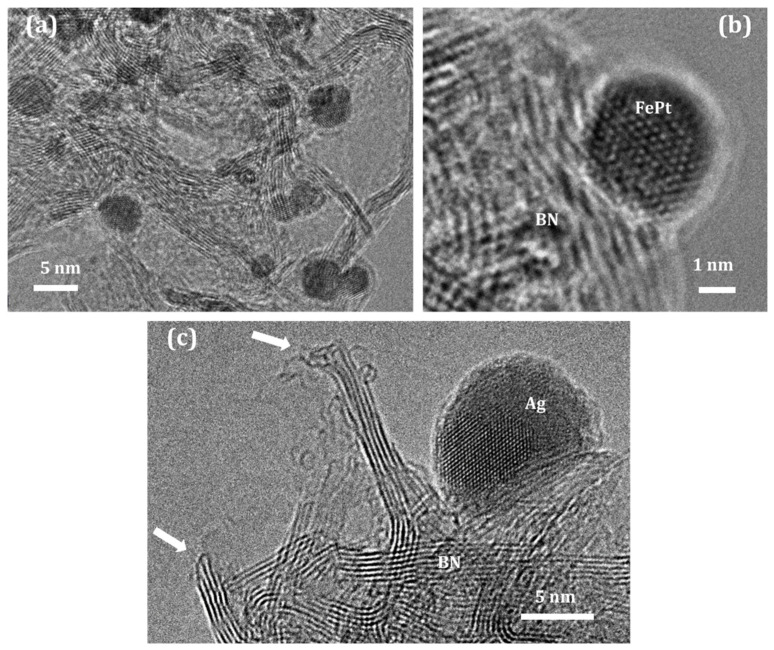
Heterogeneous FePt/*h*-BN (**a**,**b**) and Ag/*h*-BN (**c**) catalysts. Arrows show active *h*-BN edges.

Many heterogeneous catalysts contain an active phase, a carrier, and promoters. The influence of the support on the catalytic activity, selectivity, and stability of heterogeneous catalysts can be both indirect and direct. The substrate provides not only a high surface area for the active NPs (Figure 4) but also, due to the metal–support interaction, prevents the NPs from agglomeration during temperature-activated catalytic reactions. Since active NPs are usually less than 10 nm in size, a chemical interaction between the carrier surface and the atoms of the active phase often becomes noticeable. This leads to a change in the electron density distribution of the active phase (this effect is called a strong metal–support interaction (SMSI)). Finally, the support material has a direct effect on the course of catalytic reactions due to the interaction of the reaction components with the support. The *h*-BN active edges (shown by arrows in Figure 4c) can intensify the chemical interaction with the reagents. Thus, the carrier surface provides new active sites for the activation of molecules.

Although metal oxides are the most studied class of materials for many catalytic reactions, in recent years, *h*-BN has received increased attention as a support of catalytically active species. BN nanomaterials are attractive due to their large specific surface area, high resistance to sedimentation in liquid media, relatively high thermal stability and corrosion resistance, and the ability to tune their structure and properties by doping, creating defects, or by surface functionalization. The use of *h*-BN as a carrier can increase the activity of the catalyst compared to conventional supports. Due to their layered structure, *h*-BN provides a high density of attached metal NPs, which leads to increased catalytic activity [66,67]. BN has unshared electron pairs localized on nitrogen atoms, resulting in a polarized state. In addition, the electronic properties of *h*-BN depend very strongly on impurities, the introduction of which makes it possible to reduce the band gap. One of the paradoxes of *h*-BN is that, being a highly inert material, it has its own catalytic activity. This was clearly shown in the reaction of the oxidative dehydrogenation of propane [68]. The available experimental and theoretical data indicate that the *h*-BN surface acts as a driver of conversion [69,70,71].

The combination of different functional properties in one material can lead to additional advantages in the implementation of catalytic processes. For example, by creating core–shell Co@*h*-BN structures, it was possible to achieve a high activity and structural stability of Co NPs, and their ferromagnetic nature made it possible to carry out the hydrogenation of nitroarenes in aqueous media [72]. Hexagonal BN acted as a protective layer, reducing the probability of Co NP agglomeration and, as a result, contributed to an increase in the active surface and adsorption of reagent molecules. The FeNiCo/*h*-BN material was characterized as a highly active and selective catalyst for the hydrogenolysis of hydroxymethylfurfural [73]. The high BE between the FeNi and Co NPs and the *h*-BN support ensured the stability of the catalyst and the possibility of its reuse. The surface chemistry of the support in the heterogeneous catalyst significantly affects the material performance. It was shown that a large number of B-O and N-H groups in the Co/*h*-BN catalyst leads to an enhanced CoO/*h*-BN interaction, which prevents the reduction of Co oxide to active metal NPs [74]. The low amount of functional groups leads to the formation of larger Co NPs due to the reduced BE. An increased adsorption of cinnamaldehyde was associated with a high concentration of NH groups. The interaction of *h*-BN with active NPs can be enhanced by additional surface functionalization. Thus, the stability of catalytically active sites deposited on the *h*-BN surface during the oxidative desulfurization of the fuel was increased by modifying *h*-BN with an ionic liquid [75]. The modification also led to an increase in the efficiency of catalyst recirculation due to the enhanced interaction of the acid with the *h*-BN surface. The excellent catalytic properties of BN-based catalysts are largely due to the presence of defects. It was shown that the B-OH bonds formed at the edges of defective *h*-BN flakes serve as anchor centers for Cu NPs [76]. The strong interaction force resulted in a high resistance of Cu to sintering, which is usually difficult due to the low Hutting temperature of copper. The synthesized catalysts were tested in the reaction of ethanol dehydrogenation and showed a better ability to selectively form acetaldehyde compared to oxide counterparts (silica and alumina). The observed effect is explained by the difference in acetaldehyde binding: the *h*-BN-based catalysts were able to adsorb ethanol strongly, whereas the interaction with acetaldehyde was weak. At the same time, oxide-supported catalysts showed a strong interaction with both ethanol and acetaldehyde. This behavior resulted in an excellent selectivity of *h*-BN-based systems in the production of acetaldehyde.

The high catalytic efficiency of many heterogeneous materials is associated with the small size of active centers. The small size of Ag NPs and their maximum density on the *h*-BN surface were shown to be key parameters determining the high catalytic activity. Even a slight increase in the Ag NP size led to a noticeable deterioration in the catalytic performance in terms of offset and full conversion temperatures [77]. Pt cluster-supported BNNSs (with 100 ppm of Pt) were characterized as a highly efficient catalyst for propane dehydrogenation with high propane conversion (~15%) and propylene selectivity (>99%) at a relatively low reaction temperature (520 °C) [78]. To reduce the size of Pd NPs on an *h*-BN support, it was proposed to use an intermediate MgO layer [79]. It was found that, in addition to reducing the Pd size, Mg^2+^ ions partially penetrate into the *h*-BN crystal lattice, filling B vacancies. This contributed to an increase in oxygen adsorption and had a positive effect on the catalytic activity. The advantage of the metal oxide/BN interface was also demonstrated for the Ni/CeO_2_/*h*-BN system [80], in which, Ni NPs were imbedded between cerium oxide and BN. The stability of Ni NPs was increased due to the metal/support interaction, which permitted reducing coke formation in the dry methane reforming reaction. The high CO_2_ conversion rate observed on the Au/BN and Pt/BN catalysts is associated with better CO_2_ adsorption on the oxidized BN surface. A charge density distribution at the Pt/*h*-BN interface increases oxygen absorption, thereby accelerating oxygen-associated chemical reactions [81].

Fe_3_O_4_/BN, Fe_3_O_4_(Pt)/BN, and FePt/BN nanohybrids were obtained via polyol synthesis in ethylene glycol [82]. BN supporting bimetallic FePt NPs demonstrated a significantly higher CO_2_ conversion rate compared to Fe_3_O_4_/BN and Fe_3_O_4_(Pt)/BN counterparts and an almost 100% selectivity to CO, whereas catalysts with Fe_3_O_4_ NPs showed a better selectivity to hydrocarbons. An important result is the formation of core–shell *h*-BN@FePt structures upon heating, which prevents the agglomeration of catalytically active NPs.

In the case of SMSI, the metal NP can be encapsulated in a carrier material. This can have both a positive and negative effect on the catalyst performance. Hexagonal BN rarely exhibits the SMSI effect due to its relative inertness; however, BO bonds present as surface functional groups can act as SMSI sites. SMSI between FeO_x_ and BO_x_ was observed in Fe/*h*-BN catalysts, which led to the coordination of reduction and oxidation processes during the oxidative dehydrogenation of ethylbenzene to styrene [83]. As with oxide supports, SMSI can be customized with redox cycles in Pt/*h*-BN catalysts. It has been shown that the oxidative treatment of Pt/*h*-BN at temperatures above 520 °C leads to the formation of BO_x_ species and their strong interaction with metal NPs [84]. The formation of thin layers of boron oxide on top of Pt NPs was observed, which blocked low-coordinated Pt centers. Encapsulated Pt NPs were resistant to sintering, and the particle size remained unchanged during catalysis. The Pt/*h*-BN catalysts showed better stability in the propane dehydrogenation reaction compared to the Pt/Al_2_O_3_ counterpart due to the lower rate of coke formation. The surface oxygen-terminated groups of *h*-BN can lead to too strong an interaction between the active phase and the carrier and adversely affect the reducibility of metal oxide NPs. To overcome this drawback, Fe/*h*-BN catalysts doped with Cu and Mn were synthesized for the Fischer–Tropsch process [85]. Doping Fe/*h*-BN with Mn promotes the reduction and formation of active iron carbides, while Cu lowers the Fe-O reduction temperature, creating more active sites for H_2_ dissociation. The observed synergistic effect of the promoters increased the catalyst activities without reducing their stability. However, in some cases, too strong an active phase/*h*-BN interaction is unfavorable, as in the case of an acid–base catalytic reaction such as the synthesis of dimethyl ether by dehydration of methanol [86]. This reaction usually proceeds on Brønsted acid catalysts and is therefore sensitive to the number and strength of the acid sites. In this case, *h*-BN is not an ideal carrier among SiO_2_, TiO_2_, ZrO_2_, Al_2_O_3_, and CeO_2_ due to the strong interaction between tungstosilicic acid and the support, which leads to a decrease in proton mobility and catalyst efficiency.

Elemental doping is an effective approach to band gap engineering. Doping with carbon makes it possible to control the chemical activity of *h*-BN by changing the electron density near the Fermi level. Carbon-doped *h*-BN showed increased activity in the condensation of benzaldehyde with malononitrile to benzylidenemalononitrile, surpassing the undoped *h*-BN and C_3_N_4_ counterparts [87]. The high activity is explained by the decrease in the desorption barrier of the reaction products due to carbon doping. The addition of Se to *h*-BN has been shown to narrow the band gap and improve carrier generation and separation [88].

A number of studies have shown that the direct interaction of the *h*-BN support with the reaction products affects the catalytic characteristics. A comparison of Pd NP catalysts supported on *h*-BN, Al_2_O_3_, and MgO showed that the adsorption of maleic anhydride on Pd centers is improved as a result of the Pd to carrier interaction, and the Pd/*h*-BN system is characterized by increased adsorption on *h*-BN, which has a direct effect on the increase in catalytic activity [89]. The formation rate of succinic acid 6000 g/gkat/h at a selectivity of 99.7% on the Pd/*h*-BN catalyst was higher than on their oxide-based counterparts. The catalytic activity can be increased due to the formation of hydroxyl groups on the *h*-BN surface [90]. For example, an increase in the sonication time made it possible not only to increase the loading of metal, which led to an increase in the reduction rate of 2-nitroaniline, but also to increase the activity of catalysts due to the formation of hydroxyl surface groups [91]. However, in some cases, the relatively inert *h*-BN surface provides high selectivity for target products. A comparison of Pt-catalysts on various supports during the hydrogenation of cinnamic aldehyde to cinnamic alcohol showed that the absence of acid–base sites on the *h*-BN surface makes it an ideal candidate as a support for this reaction, since the process proceeds through a simple non-dissociative adsorption of cinnamic aldehyde. This results in a selectivity of over 85% towards cinnamyl alcohol. In the case of Al_2_O_3_ and SiO_2_ supports with a large number of acid–base sites, the selectivity is hindered due to the multiple-adsorption regime of the reagent.

Hexagonal BN can affect the electronic structure of deposited metal NPs. It was shown that the location of Ru on the *h*-BN edges leads to an increase in the hydrogenation activity due to the enhancement of interfacial electronic effects between Ru and the BN surface [92]. Electron-enriched NPs showed high activity in the production of primary amines from carbonyl compounds without the need for excess ammonia. For practical application, it is very important to control the electron density. An important step in this direction is the work of Zhu et al. [93], showing that the change in the electron density of Pt NPs is possible by adjusting the B and N vacancies in *h*-BN. Pt NPs are located at the site of boron and nitrogen vacancies acting as Lewis acids and Lewis bases, respectively. Thus, the synthesis of a Pt/*h*-BN nanohybrid with a predominance of nitrogen vacancies leads to an interfacial electronic effect that promotes O_2_ adsorption, reduces CO poisoning, and increases the overall activity and stability of the catalyst in the CO oxidation reaction.

The 2D morphology of *h*-BN allows it to be successfully utilized as a homogeneous catalyst in liquid heterogeneous catalytic reactions due to its good dispersibility and, hence, high specific surface area. To obtain a metal-free catalyst for the oxidative desulfurization of aromatic compounds, N-hydroxyphthalimide (NHPI) was covalently grafted to the *h*-BN surface [94]. The strong interaction between NHPI and *h*-BN results in a high catalyst activity, selectivity and recyclability. Rana et al. [95] covalently grafted a copper complex onto the surface of exfoliated BNNSs. To ensure the stability of the complex, 3-aminopropyltriethoxysilane was used as a covalent linker. Leaching tests showed that there was no depletion of Cu from the catalyst surface. The materials showed exceptional activity in the azide-nitrile cycloaddition reaction to produce the pharmaceutically important 5-substituted 1H-tetrazole.

Single-atom catalysts (SACs) that combine the advantages of homogeneous and heterogeneous catalysts are of great interest due to exceptionally high catalytic activity in a wide variety of industrially important catalytic reactions [96]. To commemorate the 10th anniversary of the introduction of the term SACs, a recent review examined various single-atom–host combinations and related applications [97]. From an economic point of view, it is highly desirable to reduce the content of expensive metals of the Pt group without compromising activity, selectivity, and stability. The chemical activity of *h*-BN can be fine-tuned using lattice defect engineering. For example, during the cryogenic grinding of *h*-BN powders, vacancies are formed that can serve as active centers for the spontaneous reduction of metal cations [98]. The authors showed the possibility of the formation of single atoms and clusters of Ag, Au, Pt, Cu, and Fe on *h*-BN defects. The nitrogen-containing B vacancy in *h*-BN can effectively anchor and confine Pd atoms [99]. Hexagonal BN was shown to be a suitable material for dispersing Cu-Pt clusters due to the abundant B-O species on its surface [78]. Nanoscale clusters (1–2 nm) were active and stable in the catalytic dehydrogenation of propane to propylene.

It is important to note that the choice of support material should be carried out taking into account the catalytic reaction mechanism. For example, the study of catalysts in the oxidative dehydrogenation of alcohols showed that the support material should be able to accept and conduct electrons between catalytically active NPs [100]. From this point of view, *h*-BN was inferior to C and TiO_2_ due to its high band gap.

Hexagonal BN also finds its application in a relatively new catalytic direction: microwave catalysis. Mo_2_C/*h*-BN nanomaterials were proposed as highly active H_2_S decomposition catalysts [101]. However, the role of the support in this area of catalytic processes is still poorly understood.

### 3.2. Photocatalysts and Electrocatalysts

Photocatalysis and electrocatalysis are alternative processes for efficient chemical transformations. In contrast to thermal catalysis, additional energy enters the system from an external source, which makes it possible to overcome the thermodynamic barrier and shift the chemical equilibrium towards the reaction products. In photocatalytic and electrocatalytic processes, materials, in addition to the ability to adsorb and activate chemical reagents, must be able to conduct electrons and be activated under the action of external energy. From this point of view, *h*-BN is not an ideal material. Due to the high band gap, a relatively high energy is required to excite an electron and transfer it from the valence band to the conduction band. However, the combination of *h*-BN with other materials proved to be effective in achieving high activity and stability.

The efficiency of the photodegradation of organic pollutants under visible light irradiation can be increased in the presence of *h*-BN. For example, the addition of *h*-BN to the ZnFe_2_O_4_ photocatalyst decreased the recombination rate of electron–hole pairs, which affected the efficiency of Congo red and tetracycline degradation [102]. The incorporation of *h*-BN into 2D Bi_2_WO_6_ flakes increased the rate of charge separation and reduced the electron–hole recombination [103]. This increased the ability to degrade antibiotics by 1.87 times. The 2D/2D *h*-BN/Bi_2_WO_6_ heterostructures also showed high activity in the antibiotic degradation [104]. MoS_2_/*h*-BN/reduced graphene oxide (rGO) composites were developed and tested for water splitting under sunlight [105]. The simultaneous presence of BN/rGO, which has a high structural stability and a large number of surface centers, and MoS_2_ with a small band gap, provides a synergistic catalytic effect. The hydrogen evolution rate of 1490.3 µmol/h/g was 58.2 and 12.2 times higher than that of the BN/rGO and MoS_2_ counterparts. The high catalytic activity and selectivity of *h*-BN-based photocatalysts in the reaction of nitrate reduction under UV radiation was explained by the formation of photoelectrons and CO_2_^−^ radicals [106]. BN provided a high concentration of adsorption sites with B atoms acting as Lewis acids, and its electronic configuration was well-suited for efficient nitrate reduction. The hexagonal BN support was shown to provide a sink of excited electrons from the surface of catalytically active Cu_3_P nanoparticles, making them active on the *h*-BN surface for the adsorption of reagent molecules [107]. BN was characterized as a promising catalyst for organic degradation via the activation of peroxymonosulfate. The defect-driven non-radical oxidation of porous *h*-BN nanorods was proposed as the main mechanism of sulfamethoxazole degradation via the formation of singlet oxygen (^1^O_2_) [108]. Used BN was easily regenerated upon heating in air, which completely recovered the B–O bonds.

a-MoS_x_O_y_/*h*-BN_x_O_y_ nanomaterial with a tuned MoS_x_O_y_ zonal structure for photoinduced water splitting was developed [109]. The nanohybrid showed high activity in the photocatalytic degradation of methylene blue (MB) (5.51 mmol g^−1^ h^−1^ under illumination with a mercury lamp), four times higher than that of known non-metallic catalysts. The obtained photocatalyst is very stable and can be reused. Oxygen-substituted BN has good wettability, and the interaction of oxygen defects with sulfur leads to the formation of S-doped BN, which has a high photodegradation efficiency when illuminated with visible light [110].

To adapt the commercial PtRu/C electrocatalyst as an anode in fuel cells with a proton-exchange membrane, metal NPs on a carbon support were encapsulated in few-layers *h*-BN [111]. The *h*-BN shell weakened the CO adsorption on the PtRu surface and increased the resistance to CO in H_2_-O_2_ fuel cells. The structural stability of Mo_2_N electrocatalysts for the nitrogen reduction reaction was improved by combining molybdenum nitride with *h*-BN [112]. The optimal catalyst exhibited a NH_3_ yield rate of 58.5 µg/h and a Faraday efficiency of 61.5%. By designing *h*-BN defects, an optimum conversion rate was achieved at a lower overvoltage. Carbon-doped *h*-BN was used to create enzyme-like single-atom Co electrocatalysts for the dechlorination reaction [113]. Locally polarized B-N bonds played a key role in the adsorption of organochlorine compounds, improving the electrocatalytic activity. No such effect was observed on carbon and graphite supports doped with nitrogen.

Heterojunctions in *h*-BN-doped BiFeO_3_ and MnFeO_3_ perovskite-based catalysts exhibited an extended visible light range, reduced band gap energy, and low recombination rate, and had excellent photocatalytic activity towards various antibiotics and dyes. The good photocatalytic performance of heterojunctions was explained by an extended excitation wavelength and a slow recombination rate of charge carriers [114].

In conclusion, we note the important role of the *h*-BN carrier, which, interacting with surface NPs, can increase their structural stability and change their electron density, which affects the catalytic activity, selectivity, and stability. Hexagonal BN can also be directly involved in chemical reactions, creating additional sites for component activation. All of these effects can have a positive or negative effect depending on the type of reaction, which is quite consistent with the well-known Sabatier–Balandin principle. Therefore, when designing a catalytic system, one should take into account the mechanisms of catalytic reactions.

## 4. Materials for Biomedicine and Improvement of Quality of Life

### 4.1. Biocompatibility and Dose-Dependent Toxicity

The biocompatibility and bio-application of BN nanomaterials have recently been reviewed [115]. Available data indicate that the biocompatibility of BN depends on the concentration, size, and shape of BNNPs. In earlier studies, the cytocompatibility of BN nanostructures was evaluated in relation to various cell cultures, such as kidneys, epithelium, human skin, ovarian, bone tissue, human carcinoma, human osteosarcoma, human lung epithelial adenocarcinoma, human neuroblastoma, and others. The results show that a concentration of BNNPs less than 40 μg/mL is safe for the vast majority of cell lines, regardless of the size (5–200 nm) and shape (nanospheres, NSs, NTs). Moreover, a low concentration of BNNPs can stimulate cell proliferation. The addition of BNNPs to differentiated NT-2 cells increased their viability by 6% (6.25 µg/mL) and 13% (3.12 µg/mL) [116]. Low BN concentrations (10–100 μg/mL) increased the viability of human umbilical vein endothelial cells by 118% (*p* < 0.05), while a NP concentration of 150 and 200 μg/mL had no significant effect on cell metabolism [106]. This effect can be explained by the influence of NPs at their low concentration on oxidative stress. Radicals (°OH, °OOH) can react with proteins, enzymes, nucleic acids, and other cell biomolecules, causing their damage and cellular apoptosis. Recently, it was shown that *h*-BN can slowly dissolve in phosphate-buffered saline (PBS) and lysosome mimicking solution, while the released boron can form boric acid [117], which exhibits antioxidant and antiapoptotic effects [118,119,120]. Boric acids and their esters are cleaved by H_2_O_2_ and other reactive oxygen species (ROS) to form the corresponding alcohol and boric acid, which are considered nontoxic to human cells [121]. The effect of BNNPs on cell viability (mHippo E-14 cells) was assessed in the presence of doxorubicin (DOX) at a concentration high enough to cause cellular stress but low enough not to kill all cells [122]. The obtained results indicate that *h*-BN reduces DOX-induced oxidative stress on cells at a concentration of 44 μg/mL.

The dose-dependent toxic effect of *h*-BN NPs was studied in vivo in albino rats (Wistar) by measuring thiol/disulfide homeostasis, lipid hydroperoxide levels, and myeloperoxidase and catalase activity [123]. After the intravenous administration of various BN doses, hematological and biochemical parameters did not change up to concentrations of 800 µg/kg. However, at doses of *h*-BNNPs greater than 1600 μg/kg, significant damage to the liver, kidneys, heart, spleen, and pancreas was observed. The results showed that *h*-BNNPs with a diameter of 120 nm are non-toxic and can be used in biomedical applications at low doses of 50 to 800 µg/kg.

### 4.2. Antibacterial and Antifungal Activity

BNNPs and BN-based nanohybrids exhibit antibacterial and antifungal activity; some recent results are presented in Table 1.

The following bacteria and fungi suppression mechanisms are noted: chemical (ROS formation [122], slow BN degradation with the formation of boric acid), structural (contact-killing by sharp BN surface structures [133] or an increase in specific surface area, leading to greater damage to bacteria [134,135]), and electrostatic (negatively charged BNNPs interact with bacterial cell walls). A contact-killing bactericidal effect of BNNPs was compared with the toxic effect of gentamicin-loaded BNNPs at the minimal inhibitory concentration of 150 mg/mL [133]. To enhance antibacterial activity, various hybrid NPs, such as BNNPs-Ag [129], BNNPs-ZnO [136], BNNPs-Cu [137], BNNPs-Zr [138], and antibiotic-loaded BNNPs [129] and films [133], have been developed. The combination of bactericidal ions and antibiotics allows one to achieve a pronounced synergistic effect. For example, gentamicin-loaded BNNPs exhibited high bactericidal activity against *S. aureus*, *P. aeruginosa*, and 38 types of the *E. coli* strains, including multidrug-resistant ones [129]. For the rest of the tested *E. coli* strains, the Ag NPs-containing nanohybrids showed superior bactericidal efficiency. In addition, Ag/BN, amphotericin B/BN, and amphotericin B/Ag/BN nanohybrids revealed high fungicidal activity.

Recently, antibiotic-like activity of *h*-BNNSs combatting antimicrobial-resistant bacteria through a Z-ring constriction damage mechanism has been reported [139]. BNNSs target key surface proteins (FtsP, EnvC, and TolB) and disrupt Z ring constructions.

### 4.3. Drug Delivery

BNNPs are promising carriers for targeted drug delivery. Since most therapeutic agents (TA) have a complex structure and/or charge, the formation of *h*-BN/TA complexes is possible due to van der Waals and/or electrostatic interactions between the components. Hexagonal BN is a good adsorbent for a large number of TA.

The high dispersibility and low reactivity of the BN surface are important factors when introducing NPs into the bloodstream to avoid thrombus formation and hemolytic activity [140]. Since the *h*-BN surface is hydrophobic and the material has high absorbent properties, surface functionalization by chemical treatment or coating with polymers is necessary. For example, stable BNNTs and *h*-BN dispersions were obtained using an O_3_-based advanced oxidation process followed by polyethylenimine functionalization [65].

The cytotoxicity of exfoliated *h*-BN flakes functionalized with hydroxyl groups (BN-OH) was studied in various models: insect hemocytes (in vivo), human erythrocytes, and mouse fibroblasts (in vitro) [141]. Long-term immunoassays showed that BN-OH, despite the absence of hemocytotoxicity, impaired nodulation, the most important cellular immune response in insects. Hemocytes exposed to BN-OH and then to bacteria differed in morphology and adhesiveness from hemocytes exposed to bacteria alone and exhibited the same morphology and adhesiveness as control hemocytes. Thus, the BN-OH-induced decrease in nodularity may be the result of a decrease in the ability of hemocytes to recognize bacteria, migrate to them, or form microaggregates around them, which can lead to immune system dysfunction when infected by pathogens. Long-term in vivo studies are still needed to unambiguously confirm that *h*-BN is biocompatible and can be utilized as a drug delivery platform or for bioimaging.

In addition to surface functionalization, which improves dispersibility and biocompatibility, the attachment of specific surface ligands or complexes is necessary to ensure the selectivity of drug delivery carriers. Using the self-assembly approach, a-BN-based hybrid NPs containing DOX and a conjugate of a mixture of folic acid (FA) and chitosan (CH) were prepared [142]. N-(3-dimethylaminopropyl)-N′-ethylcarbodiimide hydrochloride was used as the crosslinking agent. The DOX-loading capacity in a-BN with and without FA-CH was 7.52 ± 0.32% and 6.24 ± 0.58%, respectively. For in vivo cell visualization, DOX was replaced with cyanine 5.5 as a label. Mice injected with a-BN-Cy5.5@FA-CH showed intense fluorescence in the tumor region, indicating the effectiveness of FA-CH conjugates for targeted drug delivery.

For the treatment of the most aggressive type of brain cancer (glioblastoma multiforme), BNNTs were coated with cell membranes extracted from glioblastoma cells [143]. Effective targeted drug delivery is based on the homotypic recognition of tumor cells included in the carrier. The cell membrane-conjugated BN complexes acted specifically and effectively killed cancer cells without affecting healthy brain cells.

BN nanostructures are also being used to create theranostic systems with simultaneous therapeutic and diagnostic capabilities. For example, *h*-BNNSs were used to create a multifunctional theranostic platform containing DNA oligonucleotide and copper (II) phthalocyanine (Pc) [144]. The nanohybrids showed the effective drugs accumulation in tumor cells and remarkable photodynamic therapy efficiency with minimized damage to normal tissues. The Cu Pc molecule was used as a photosensitizer in photodynamic therapy and as a sensitive and accurate diagnostic probe for the in situ monitoring and visualization of miR-21 by surface-enhanced Raman spectroscopy.

### 4.4. Boron Neutron Capture Therapy

Natural elemental boron contains approximately 20% stable Boron-10 isotopes sensitive to neutron irradiation and 80% Boron-11 isotopes [145]. Boron neutron capture therapy (BNCT) is an emerging and non-invasive cancer treatment strategy based on the selective accumulation of boron compounds in tumor cells and subsequent exposure to a thermal neutron beam. The fundamentals of BNCT and clinical applications were recently reviewed by Malouff et al. [146]. Note that clinically approved B-containing drugs (boronophenylalanine and mercaptoundecahydrododecaborane) have a low cumulative capacity and high cost [147].

BNNPs are promising boron carriers containing 50% B (B-10 or B-11) for BNCT. The main challenge is to achieve sufficient boron accumulation in tumor cells and ensure the subsequent degradation of nanocarriers in order to avoid in vivo toxicity. To help solve this important problem, BNNPs were coated with phase-transitioned lysozyme, which protects nanocarriers from hydrolysis during circulation in blood and can be readily removed with vitamin C after BNCT [148]. The effectiveness of tumor treatment can be enhanced by a combination of antibiotics and neutron therapy. The tumor size decreased by 48.2 ± 17.0% (DOX/BNNSs), 64.1 ± 10.9% (BNNSs + neutron irradiation) and 94.6 ± 2.1% (DOX/BNNSs + neutron irradiation) [149]. In addition, BN nanostructures can be further doped or functionalized to endow them additional functionality. For example, ^64^Cu radioisotope was added to BNNTs as a biological marker for diagnostic purposes [150]. Cu as a chelating element can potentially bind to peptides and other important biological molecules, such as antibodies, proteins, and nanoparticles.

### 4.5. Tissue Engineering

BN nanostructures can be used to strengthen tissue-engineering scaffolds and stimulate cell adhesion and proliferation for efficient tissue regeneration. The addition of BNNTs to tricalcium phosphate scaffolds not only increased mechanical properties but also enhanced bone morphogenetic proteins, the osteogenic differentiation of mesenchymal stem cells, Runx2 expression, and new bone formation [151]. The doping of porous BN nanofibers to poly(vinyl alcohol) (PVA) hydrogels was shown to improve its mechanical properties, swelling ability, and thermal stability [152]. Exfoliated BN was used as filler in 3D porous polylactic acid (PLA) scaffolds. The addition of 0.1% BN to PLA scaffolds increased the polymer degradation temperature from 298 °C to 359 °C and improved the swelling from 80 to 118 [153]. The presence of BN in 3D-printed PLA/BN scaffolds did not affect the viability of MG-63 and MC3T3-E1 cells (MTT assay) after 4 or 7 days. Adhesion, proliferation, and cell mineralization on PLA/BN composites were higher compared to the BN-free counterpart.

PVA/*h*-BN/bacterial cellulose scaffolds for bone tissue engineering were fabricated using 3D printing [154]. The obtained composites showed 100% human osteoblast cell viability after 72 h incubation. The *h*-BN was used as a filler due to its superior thermal and mechanical properties. The addition of 10% BNNPs to Poly(N-methylpyrrole) increased the antibacterial effect against four types of strains (*Escherichia coli*, *Staphylococcus aureus*, *Pseudomonas aeruginosa*, and *Enterococcus faecalisbacterial*) [124]. Kirschner wires were coated with *h*-BN films by magnetron sputtering and studied in the treatment of fractures in adult male Wistar albino rats [155]. According to microcomputed tomography, BN-coated implants showed better healing characteristics compared to the control group.

A comparison of the physicochemical properties of a bioceramic-based root canal sealer used in endodontics reinforced with various nanomaterials (multi-walled carbon nanotubes (CNTs), titanium carbide, and *h*-BN) showed that the BN-doped composites have minimal initial (at 1%BN) and final (at 2%BN) setting times [156]. The potential use of BNNPs for wound healing was studied using human umbilical vein endothelial (HUVE) and human dermal fibroblast (HDF) cells and compared with boric acid, which is one of the degradation products of *h*-BN [117]. The proliferation and migration of HUVE and HDF cells were significantly higher in BN-treated cultures compared to boric-acid-treated cells. Hexagonal BNNPs showed no cytotoxicity at concentrations of 25–200 μg/mL. Composites based on chitosan polyhydroxyalkanoates doped with BNNPs (0.1–1.0%) were obtained using a simple solvent casting technique [126]. Compared to a negative control, the addition of BNNPs reduced the percent viability of *E.coli* K1 and multidrug-resistant *S. aureus* strains.

### 4.6. Face Masks

COVID-19 has increased the demand for long-term surgical masks. To help solve this important problem, commercial polypropylene nonwovens have been functionalized with *h*-BNNPs [127]. NPs-modified fibrous membranes not only inhibited 99.3% and 96.1% of *E. coli* and *S. aureus* strains, respectively, through a contact killing mechanism, but also significantly increased thermal conductivity and heat dissipation.

### 4.7. Biosensors

A number of works were devoted to the use of *h*-BN, both alone and as a part of heterogeneous nanostructures, as biosensors for detecting small amounts of organic substances. BNNSs exhibited an excellent adsorption of pharmaceuticals [157]. It was shown that the formation of a chemical bond between the hydrogen atom in the drug and the nitrogen atom in BN promotes good adsorption. The encapsulation of C60 fullerene in BNNTs led to an increase in the sensitivity level of the nanoprobe up to several hundred molecules [158]. This is due to the enhancement of the near-field vibrational spectra of the analyzed molecules near the nanotube surface due to phonon–polariton excitation, which is close to molecular. There are several reports on the use of *h*-BN QDs as a biosensing platform; in particular, for the electrochemical detection of ascorbic acid [159], vitamin C [160], and dopamine (a neuromodulatory molecule that plays an important role in cells) [161]. To detect FA, electrochemiluminescence biosensors were developed based on the effect of resonant energy transfer between QDs [162]. In such biosensors, BN QDs and N-doped Gr QDs acted as a donor and acceptor, respectively. The addition of BN QD resulted in an approximately 10-fold signal amplification. The proposed sensor showed a wide linear range from 1.0 × 10^−11^ M to 1.0 × 10^−4^ M and a low detection limit of 5.13 × 10^−12^ M. Recently, the use of *h*-BN QDs as an electrochemical sensor for ferritin (an iron-storing protein) has also been reported [163].

### 4.8. Sponges and Membranes for Water Purification

The adsorption properties of the material can be divided into physical and chemical adsorption. During physical adsorption, interactions between adsorbents and adsorbates are determined by van der Waals forces and electrostatic interactions. During chemical adsorption, chemical bonds can form between the surface of the adsorbent and adsorbate [164]. Due to the porous structure with numerous structural defects and the presence of B-N ionic bonds with coexisting basic sites (N atoms) and Lewis acid sites (B atoms), *h*-BN materials are the subject of many studies aimed at developing new materials for purifying liquid media from various contaminants: dyes, organic compounds, antibiotics, and heavy metals. The latest development in BN-based materials in water purification are considered in a recent review [165].

BNNPs have a high adsorption capacity for MB and Rhodamine B (RhB) dyes; their maximum adsorption capacities were 330.00 and 302.50 mg/g, respectively [166]. To increase the adsorption capacity for dibenzothiophene, BNNPs were doped with carbon [167]. BCN materials showed a better adsorption capacity than pure *h*-BN, which is associated with a reduction in chemical hardness according to the theory of hard and soft acids and bases. The catalytic activity of BNNPs doped with cobalt was studied with respect to the MB dye [168]. When using the BNNPs-10%Co material, a 99% dye degradation was observed in just 1 min. BN nanomaterials show a high degree of reuse. For example, the efficiency of the adsorption of MB molecules from water by BNNTs was approximately 94%, even after three cycles of use [169].

Due to its large specific surface area and hydrophobic properties, porous *h*-BN nanomaterials have attracted much attention as sponges and membranes for adsorbing organic solvents, medicals, hydrocarbons, and oils from water. When using enzyme-immobilized BNNSs, hydrocarbon degradation by 89% was achieved after 72 h (pH 7), and the efficiency decreased only to 54% after five reuse cycles [170]. Highly porous BNNSs with B vacancies showed an improvement in the sorption capacity with respect to tetracycline by 38% compared to the pristine BNNSs [171]. Silane-impregnated *h*-BN-based sponges with superhydrophobic properties, a high oil adsorption, and superior stability in acid, alkali, and salt environments have been obtained [172]. In addition, these materials show an enhanced fire resistance and high compressive strength.

One promising strategy for water purification is the incorporation of BNNPs into a polymer mat. Nanocomposite membranes with *h*-BN additives were obtained by phase inversion, polymerization, deposition, filtration, and electrospinning [165,173]. BNNPs were added to poly(arylene nitrile) [174], polytetrafluoroethylene [175], polysulfone [176,177], and cellulose ester [178] membranes. These systems have demonstrated a high efficiency in separating various oil/water emulsions [174], desalination [179], and the removal of water-soluble dyes such as methyl orange, methylene blue, Evans blue [175], acid blue, and bromophenol blue [176]. The 2D multilayer structure of the BNNS contributes to the improvement in water transport and the selectivity of molecular permeation [177]. BNNPs were also added to anodized aluminum membranes [180] and stainless steel wire cloth [181] to desalinate hypersaline water. The *h*-BN nanoflake-based membranes effectively removed various anionic dyes and salts while maintaining a stable water permeability [180].

Salinity gradient energy is a clean and renewable source of energy. For the reverse electrodialysis process, obtaining separation membranes with a superior performance for harvesting osmotic energy remains a challenge. To address this important problem, BNNSs were introduced into Ti_3_C_2_T_x_ MXene membranes to create 2D laminar nanochannels [182]. This modification significantly reduced the membrane internal resistance and increased the output power density to 2.3 W/cm^2^ (44 wt% BN).

### 4.9. Textile Materials

The modern textile industry actively uses nanotechnology in the production of fabric materials. The addition of nanomaterials to the fabric composition make it possible to control various properties, such as strength, elasticity, electrical conductivity, hydrophobicity, UV protection, heat resistance, self-cleaning, antibacterial activity, etc. BNNPs have been utilized to modify the surface of cotton fabric to impart high heat and flame resistance [183,184]. The pretreatment of BNNPs with various organic compounds containing functional groups allows one to increase the adhesion strength of particles to fabric, improve their distribution over the support surface, and increase the heat resistance and stability of textile materials. For example, fabric treatment with BNNPs in a solution containing 4% Hercosett XC resin and 1.2% 3-(N,N-dimethylmyristylammonio)propanesulfonate increased its heat and fire resistance [185]. Membranes based on cellulose nanofibers with 70 wt% of BNNSs showed a 400% improvement in thermal conductivity compared to BN-free material [186].

## 5. Composites

### 5.1. Metal Matrix Composites

**Figure 5:**
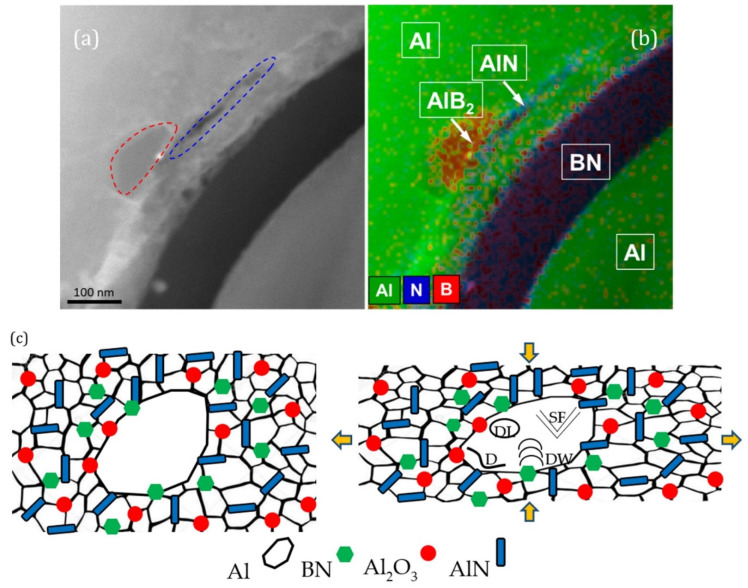
Formation of AlB_2_ and AlN phases during SPS process. HAADF-STEM micrograph (red dash line outlines AlB_2_ phase and blue dash line outlines AlN phase) (**a**) and corresponding spatially resolved EDXS map (**b**). Adapted with permission from Ref. [190]. Copyright 2019, Elsevier. Schematics of a two-level structure (**c**) consisting of micron Al grains surrounded by a composite material with nanosized Al grains and reinforcing nanosized AlN, Al_2_O_3_, and *h*-BN inclusions, which simultaneously provide high strength and ductility. D—Dislocation, DL—dislocation loop, SF—stacking fault, DW—dislocation wall. Adapted with permission from Ref. [187]. Copyright 2022, Elsevier.

Metal matrix composites (MMCs) suffer from softening at elevated temperatures, which hinders their practical application. Another problem is that it is difficult to increase the material strength without sacrificing ductility. Therefore, when developing new MMCs, the main attention is paid to increasing the strength at room and elevated temperatures while maintaining the high plasticity inherent in metals. Most of the research is devoted to composite materials with an Al matrix. BN nanostructures, such as nanotubes, nanosheets, nanoflakes, nanopellets, and nanoparticles, are being actively studied as a strengthening additive to various metals. In addition to the BNNPs making their own contribution to the strengthening, BN can react with Al at high temperatures to form multicomponent solid solutions and/or secondary phases, such as AlN and AlB_2_ (Figure 5a,b), through the following chemical reaction:

3Al + 2BN → 2AlN + AlB_2_ (1)

As-synthesized BN nanostructures often contain oxygen. A thin alumina layer covering the Al powder provides additional oxygen. Thus, oxygen can also be involved in the reactions to form the Al_2_O_3_ phase as follows [187]:

7Al + 6BNO → AlB_2_ + 6AlN + 2B_2_O_3_ (2)

2AlN + B_2_O_3_ → Al_2_O_3_ + 2BN (3)

2Al_2_O_3_ + B_2_O_3_ → 2Al_2_O_3_·B_2_O_3_ (at 800 °C) (4)

2Al_2_O_3_·B_2_O_3_ + 2AlN → 3Al_2_O_3_ + 2BN (at 1550 °C) (5)

The formation of reinforcing ceramic phases in situ as a result of chemical reactions between the system components leads to a high adhesion strength of the metal/ceramic interfaces, which ensures an enhanced mechanical strength. Earlier work on Al/*h*-BN composite engineering can be found in a recent review [188].

Aluminum matrix composites (AlMCs) reinforced with 1 wt% of BNNSs exfoliated during the ball milling of micron powder in ethylene glycol showed a room-temperature tensile strength of 152 MPa, which is 69% higher than that of the pristine Al [46]. The composites demonstrated a superior adhesion strength between Al grains and BN layers and withstood high-applied loads. Calculations showed that the critical shear stress of the AlB_2_/*h*-BN and AlN/*h*-BN interfaces is higher than that of Al/*h*-BN [189]. This indicates that the formation of AlN and AlB_2_ phases enhances the interface strengths and the strength of the entire composite. Density functional theory (DFT) calculations also showed that, due to the lower migration barrier, B atoms can diffuse more easily in the Al crystal lattice compared to N atoms, which leads to the formation of the AlB_2_ phase inside the Al grains. It is energetically more favorable for N atoms to segregate at the Al interfaces, forming the AlN grain boundary phase [190].

Gr and *h*-BN have similar crystal lattice parameters and a high tensile strength and thermal conductivity. It was shown that the addition of 0.15 wt% GrNPs to the Al-1 wt%BN composite increases the ultimate compressive strength from 168 MPa to 260 MPa [191]. BNNPs and GrNPs located at the Al grain boundaries blocked the movement of dislocations, thereby improving the mechanical properties.

The modern trend in the production of metal matrix composite materials by powder metallurgy methods is the use of nanopowders. The Al microparticles are coated with a thin oxide layer of several nm, whose thickness in NPs can increase to 6–10 nm, which introduces additional oxygen into the system. The use of AlNPs resulted in an almost threefold increase in strength compared to Al prepared using micron-sized AlNPs due to the formation of Al_2_O_3_ inclusions [187]. The further addition of 2 wt% *h*-BN nanoflakes led to an increase in tensile strength to 405 MPa (25 °C), 300 MPa (300 °C), and 240 MPa (500 °C), i.e., the increase was 82%, 64%, and 65% compared to pristine Al. The compressive strength was approximately 500 MPa (25 °C), 346 MPa (300 °C), and 200 MPa (500 °C), i.e., the increase was more than 200%. It is important to note that a high strength was achieved at a decent elongation to failure (>10%), which is explained by the formation of a two-level microstructure consisting of NPs-free Al grains surrounded by a network of metal matrix composite consisting of fine metal grains and ceramic-reinforcing NPs (Figure 5c).

The effect of *h*-BN additives on the dispersion strengthening of Al alloys has also been studied. Al7150-based composites were obtained by casting with stirring by adding BNNPs to the melt at 800 °C [192]. The addition of 1.5 wt% BN increased the tensile strength from 141 to 255 MPa and the hardness from 151 to 180 HV. It is assumed that strengthening is associated with grain refinement, but the reason for this is unclear, since BN was not observed in the composite. In Al7075/BN composites obtained by the same method, only boron was observed instead of BN [193]. Al2014- and Al7075-based composites loaded with 1, 3, and 5 wt% *h*-BN flakes were successfully fabricated using a combination of high-energy ball milling and spark plasma sintering (SPS) from powder mixtures of individual elements [194]. Composites reinforced with 3 wt% BN showed the best thermomechanical properties: the ultimate tensile strength was 310 MPa at 25 °C and 180 MPa at 500 °C (Al7075-3%BN) and 225 MPa at 25 °C and 220 MPa at 500 °C (Al2014-3%BN). It is worth noting that strain values were >8% over the entire temperature range. Composites, in addition to unreacted BNNPs, contained various reinforcing phases formed as a result of the interaction of BN with Al, Si, and Mg. The excellent mechanical properties are due to a combination of the high thermal stability of the reinforcing inclusions, solid solution strengthening, and Orowan (precipitation) strengthening. Thus, the formation of a multicomponent dispersion system with a hierarchical particle size distribution is a new strategy for metal strengthening.

Recently, the mechanical properties of Ni have been greatly improved by the addition of an ultra-low amount of *h*-BN nanoflakes (0.05 wt%), resulting in Ni grain refinement, solid solution hardening, and grain boundary strengthening [195]. Compared to Ni, the tensile strength was increased by 26% (25 °C) and 63% (750 °C) and the bending strength by 121%. The BN-doped Ni showed a high strain (>20%) in both tension and bending, and a high resistance to plastic deformation in compression.

It is also worth mentioning a recent example of obtaining AlMCs by the additive method. A dense AlSi10Mg-1 wt%BN composite reinforced with the *h*-BN, AlN, and AlB_2_ phases was obtained using selective laser melting [196]. When adding 1 wt% of *h*-BN, the phase hardness and tensile strength increased by 32% and 28%, respectively. The ceramic additive changed the heat transfer during the melting process, which affected the geometrical parameters of the melting pool and the porosity of composite.

### 5.2. Oxidation and Corrosion Protection of Metals and Alloys

Ultrathin (2–5 nm) *h*-BN films were used as high-temperature oxidation-resistant coatings to protect Cu foil up to a temperature of 500 °C and nickel and stainless steel up to 1100 °C from oxidation [197]. A small addition of *h*-BN (3–6 wt%) also makes it possible to increase the resistance of the Ti matrix to oxidation [198]. The oxidation protection mechanism is that, in laser additive manufacturing, BN reacts with Ti to form TiN and TiB phases that nucleate at grain boundaries and passivate the most reactive parts of Ti. The introduction of *h*-BN into Inconel718 increased the heat resistance of composites obtained by the laser powder bed diffusion method by blocking heat inside the matrix [199]. Thin *h*-BN films were used to protect stainless steel from a corrosive environment. Magnetron sputtered *h*-BN films showed an excellent oxidation resistance at 600 °C in air and superior corrosion resistance in a 3.5 wt% NaCl solution [200].

### 5.3. Ceramic Matrix Composites

Hexagonal BN is also used as an additive in ceramic materials. The addition of *h*-BN to (Zr,Nb)B_2_ ceramics reduced the grain size, increased the hardness and toughness, and improved the oxidation resistance [201]. Ceramics, consisting of superhard TiB_2_ grains and soft *h*-BN inclusions, were developed to improve the material resistance to thermal shock [202]. Crack propagation was hindered by crack tip blunting in soft *h*-BN inclusions.

### 5.4. Polymer Matrix Composites

Hexagonal BN is often used as a filler to improve the thermal conductivity of polymer composites. Appropriate *h*-BN surface functionalization can improve the chemical and mechanical properties of polymer composites by forming strong chemical bonds at the polymer matrix/filler interface [203]. Two-dimensional amino-modified *h*-BN was used as a laminar cross-linking agent to reduce graphene oxide wrinkling and improve its in-plane thermal conductivity [204]. The main challenges are to ensure a uniform and directed distribution of *h*-BNNPs. Recently, an original solution has been proposed for obtaining epoxy resin-based composites with a uniform distribution of *h*-BNNSs [205]. Using an external magnetic field, ZnFe_2_O_4_-decorated *h*-BNNSs [205] and Fe_3_O_4_-decorated *h*-BN platelets [206] were well-oriented in one direction. A high in-plane thermal conductivity was achieved in Ag/*h*-BN composites, in which, *h*-BN nanoplatelets were oriented along the planar direction, and Ag nanowires bridged the BN phase, forming a heat-conducting network [207].

To achieve maximum thermal conductivity, various highly porous 3D *h*-BN nanostructures filled with a polymer were developed. Recently, a 3D thermal conducting network based on *h*-BN structures was obtained, in which, BN microspheres served as the main building blocks and BNNSs were placed between them to improve the heat flux density [208]. Compared to epoxy resin, the thermal conductivity of the BN/epoxy composite with a filler content of 30 wt% increased five times (1.15 Wm^−1^K^−1^). The biaxial stress applied in the manufacture of polystyrene-based foam promotes the alignment of BNNSs and the formation of a 3D interconnected filler [209]. At 30 wt% BN, a 97% increase in thermal conductivity (up to 1.28 Wm^−1^K^−1^) was achieved. The thermal conductivity of an epoxy composite with 75 wt% of surface-modified spherical BNNPs was 62 times higher (11.8 Wm^−1^K^−1^) than that of filler-free epoxy [210]. The addition of 83 wt% *h*-BN with an ultrahigh aspect ratio to the polyvinyl alcohol matrix led to a record increase in thermal conductivity to 67.6 Wm^−^^1^K^−^^1^ [42].

### 5.5. Magnetic Composites

The development of controlled magnetic BN-based NPs is a promising approach to the obtaining of new advanced materials for various applications, such as biomedicine, electronic devices, and polymer-based composites. This goal can be achieved by using both the intrinsic magnetic properties of *h*-BN and by forming BN-based heterostructures using magnetic NPs as a system component.

Layered BNNSs (~1.5 nm thick) were reported to exhibit pronounced ferromagnetic properties [211]. Exfoliation was carried out by treating the *h*-BN powder in an ammonium fluoride solution at 180 °C for 24 h. The fluorination of *h*-BN caused spin polarization of the F and N electrons, resulting in enhanced magnetic properties with a coercive force value of approximately 100 Oe. Using the fluorination, the *h*-BN band gap was reduced and the charge density of N atoms was changed, which led to the appearance of anomalous spin-glass-type magnetism in 2D-layered materials [212]. A transition from a diamagnetic to ferromagnetic behavior of *h*-BN was reported after high-energy ball milling [213]. The saturation magnetization increased as the processing time was increased. This is due to the formation of N defects and the associated decrease in the band gap, which is accompanied by an increase in the total magnetic moment. It is assumed that the corresponding spin–split bands near the Fermi energy are responsible for the pronounced material ferromagnetic behavior. The tunable magnetic properties of single-layer *h*-BN were theoretically predicted and experimentally verified [214]. Oxygen-doped BN, prepared by reacting boric acid with a tertiary amine, exhibited a band gap reduction to 2.1 eV and paramagnetic properties. This is associated with the substitution of N atoms for oxygen and the sp^2^ type hydroxylation at the BN plane edges. An increased coercive force above 350 Oe was observed for highly defective oxygen-doped *h*-BN [215]. The pyrolysis of melamine and boric acid was used to synthesize the material. The low crystallinity and covalent doping with oxygen resulted in more unpaired electrons in the system and spin polarization, which explains the superior magnetic properties of BN.

Single-layer *h*-BN was used as a sublayer for the growth of magnetic metal NPs. Co NPs synthesized by electron beam evaporation on the *h*-BN/Pt(111) substrate surface exhibited ferromagnetism at room temperature with perpendicular magnetic anisotropy [216]. The increase in the orbital magnetic moment is associated with orbital hybridization between *h*-BN π-orbitals and Co d-orbitals at the interface.

The ferromagnetic behavior of *h*-BN was achieved by doping with Gr QD [217]. The formation of C-N and C-B bonds led to spin polarization and charge asymmetry, which provided a saturation magnetic moment of 0.033 emu/g at room temperature. The practical implementation of *h*-BN in magnetic sensor devices has been reported [218]. The *h*-BN/Gr/*h*-BN heterostructures consisting of 2D *h*-BN and Gr layers were obtained by layer-by-layer CVD transfer. The obtained Hall sensors worked reliably in the ambient environment for more than 6 months, with a sensitivity in the range of 60–97 V/A × T.

The Fe_3_O_4_/BN composites obtained by the solvothermal method were characterized as an efficient catalyst for the glycolysis of polyethylene terephthalate (PET) [219]. In addition to an increased recyclability, this heterogeneous material showed an increased catalytic activity, with a 100% conversion of PET to valuable bis(hydroxyethyl)terephthalate compared to single Fe_3_O_4_ and *h*-BN. Ni shells were obtained on the *h*-BN surface using the solvothermal method. The composite ferromagnetic properties were associated with the presence of Ni and used for the magnetic catalyst separation. The core–shell Ni@*h*-BN composite proved to be an effective catalyst for the production of hydrogen by the hydrolysis of ammonia borane, with a turn over frequency (TOF) value of 4.1 mol_H2_mol^−1^_Ni_min^−1^ [220].

Recently, heterogeneous FePt/*h*-BN NPs with pronounced magnetic properties and the ability to purify water from organic pollutants have been reported [221]. The effect of NP size stabilization was achieved by wrapping *h*-BN atomic layers around FePt NPs to form core–shell FePt@*h*-BN NPs. The temperature-activated ordering process in ultrafine FePt NPs, which is facilitated by the NPs-to-hydrogen interaction, significantly improved the magnetic properties of FePt/*h*-BN heterostructures.

Due to its good adsorption properties and high chemical inertness, *h*-BN is a promising material for wastewater treatment. Decorating Co NPs imparts ferromagnetic properties to the BNNPs, which increases the possibility of reusing the material after its separation from an aqueous solution by conventional magnetic treatment [168]. The coercive force and remanence increased with an increasing Co concentration and reached 69.4 Oe and 2.95 emu/g, respectively, for 7.5 wt% Co. Magnetic FeO_x_/*h*-BN adsorbents have shown their efficiency and high sorption capacity (up to 53 g per gram of sorbent) in the extraction of crude oil [222]. Oil adsorption occurs on *h*-BN macropores due to the effect of capillarity. The magnetic adsorbent can be easily recovered and the resulting oil can be reused. BN nanostructures can be used in membrane technology to confine magnetic ionic liquid in nanochannels formed by BNNSs to align magnetic anions that accelerate CO_2_ separation and achieve a CO_2_ permeability of ~227 Barrer [223].

Iron oxide NPs were used to decorate the *h*-BN surface in order to align them in a polymer composite under the action of a magnetic field [224]. The resulting nanocomposite exhibited a more than 400% higher dielectric constant and lower dielectric losses compared to the FeO_x_-free counterpart. The magnetic-field-controlled alignment of BNNPs has also been used to increase the thermal conductivity of thermoplastic polyurethane elastomer [225]. To achieve this, paramagnetic Fe_3_O_4_ NPs were deposited on *h*-BN. The dispersibility of the Fe_3_O_4_/BN composite was increased by surface functionalization with 2,4-toluene diisocyanate. This also made it possible to increase the affinity of additives to the polymer matrix, facilitating their ordering in a magnetic field. The thermal conductivity of the nanocomposite was 5.15 Wm^−1^K^−1^.

Hexagonal BN is also utilized to produce soft magnetic composites. The addition of BN to FeSiAl reduced the magnetic loss at 30 MHz, allowing for the use of FeSiAl/BN composites at a higher frequency than other FeSiAl-based materials [226]. As a component of heterostructures, *h*-BN has been used for electromagnetic interference protection [227]. Heterostructures based on MXene and *h*-BNNSs were fabricated by ultrasonic mixing. The addition of *h*-BN resulted in an almost doubling of thermal conductivity, a high electrical conductivity of 57.67 S/cm, and an EMI shielding efficiency of 37.29 dB.

## 6. Optical and Optoelectronic Devices

### 6.1. Quantum Dots, Single-Photon Emitters, and Devices

Blue-green QDs in *h*-BN with a quantum yield of 2.5% were first reported in 2014 [228]. Despite significant subsequent progress in this field, the lack of data on long-wavelength photoluminescence and stability limits the use of *h*-BN QDs in electronic and optoelectronic devices. Recently, thermostable BN QDs with full-color emission (420–610 nm) and a high quantum yield of 32.27% were reported [229]. The resulting optical films based on BN QDs have a multicolor display, high transparency, and good flexibility.

QDs in *h*-BN were created by etching and oxidation in orthophosphoric acid. It is generally believed that hydroxyl groups in *h*-BN are responsible for the emission in the visible range. Another new emission observed in the UV range was attributed to a characteristic infrared-active vibration [230]. The localization of a quantum emitter in all three dimensions was recently demonstrated [231]. The use of the monolayer approach made it possible to either completely suppress or activate the emission. The resulting sandwich structure also enables external emission control.

Single photon emitters (SPEs) from *h*-BN defects were discovered in 2016 [232,233,234]. BN defects have long-lived non-radiative metastable states. These quantum emitters can be switched from dark to bright mode by co-excitation photocontrol [235]. Two possible mechanisms for the observed phenomenon have been proposed: optical pumping and refilling. Due to the presence of bright and stable single-photon sources, such as impurity single atoms, QDs, and fluorescence atomic defects, 2D and 3D *h*-BN nanomaterials find many applications in quantum optics and fluidic and sensing devices [232,236]. In addition to traditional materials, such as semiconductors, chalcogenides, and carbon materials, *h*-BN of various morphologies is a promising nanomaterial for SPEs used in integrated quantum photonics [237,238,239,240,241,242].

The main advantages of SPEs in *h*-BN include an exceptional brightness, photostability, narrow linewidth, high reliability, and the ability to operate at room temperature [232,233]. Various approaches were used to create BN defects. In addition to intrinsic defects, additional defects can be created by doping, irradiation with a pulsed laser and a focused ion beam, exfoliation, mechanical processing, and high-temperature annealing ([243] and the works cited there). It has been shown that vacancy defects in *h*-BN wrinkles generate a large number of SPEs (much more than on a flat surface), with dipoles oriented in the wrinkle directions [244].

The *h*-BN monolayer on a metal substrate is an excellent template for growing cluster arrays of the same size. A quasi-ordered network of small Si clusters was successfully created on the *h*-BN surface [245]. Such materials can find application in catalysis and electronics. Optical thermometry based on quantum emitters in *h*-BN is a new approach to the monitoring of local temperatures [246]. Compared to other 2D materials, *h*-BN has a number of advantages: good thermal contact, high brightness of quantum emitters, high sensitivity, and spatial resolution over a wide temperature range (0–800 K).

Recently, much attention has been paid to the theoretical and experimental study of the fundamental properties of excitons in *h*-BN [247,248,249,250]. The indirect nature of the strongly bound lowest-energy exciton and the presence of a direct exciton at a slightly higher energy are elucidated. Experiments on the hydrostatic compression of *h*-BN made it possible to understand the different nature of intralayer and interlayer interactions in *h*-BN and to control the bandgap [251]. Phonon polaritons are capable of tightly localizing light at deep subwavelength scales. Recently, direct evidence has been obtained for the existence of phonon polaritons in the *h*-BN monolayer [252]. Phonon polaritons exhibit a high confinement (the wavelength is more than 487 times shorter than that of light in free space) and an ultraslow group velocity of up to approximately 10^−5^ s. The operation of many heterostructures used in 2D optics is based on strong exciton–phonon and exciton–electron coupling. It was shown that an inactive (dark) intervalley exciton up-converts light into an active (bright) intravalley exciton in *h*-BN-encapsulated WSe_2_ monolayers [253]. The excitation spectra show two additional lines at 34.5 and 46.0 meV below the bright exciton, which can be associated with up-conversion processes.

The concept of elemental doping has proven itself in the development of *h*-BN-based optoelectronic devices [254]. For example, doping with sulfur narrows the band gap of 2D *h*-BN and increases the material conductivity up to 1.5 times. In addition, the response wavelength of the sulfur-doped *h*-BN photodetectors was extended from 260 to 280 nm, and the photocurrent and responsivity increased by approximately 50 times when irradiated with light at a wavelength of 280 nm. The presence of different defects (vacancies and impurities) in *h*-BN causes the appearance of point color defects, which are attractive as SPEs [255,256]. Color point defects created in *h*-BN by ozone thermal treatment exhibit bright (≈1 MHz) and stable room temperature light emission with a peak zero-phonon line energy at approximately 2.16 eV [257]. The optically observed magnetic resonance in *h*-BN was attributed to the negatively charged boron vacancy, V_B_^−^ [258].

Disordered heterojunctions between atomically sharp Gr and *h*-BN were demonstrated to be sources of blue emission [259]. The emission probably originates from localized energy states at the Gr/*h*-BN interface. Rare-earth-doped *h*-BN-based anophosphors synthesized by high-temperature pyrolysis (1200 °C) exhibited blue-yellow emission activated by inserted Ce3^+^ and Dy3^+^ ions under UV irradiation [260]. The emission color can be customized by adjusting the [Dy3^+^]/[Ce3^+^] ratio.

### 6.2. Photodetectors

The presence of a wide bandgap and long-term stability when exposed to UV radiation makes it possible to use *h*-BN in deep ultraviolet photodetectors. However, *h*-BN photodetectors suffer from a relatively poor performance with a low responsivity [261]. To overcome this shortcoming, *h*-BN was doped with C, which improved its photoresponsivity by an order of magnitude [262]. The sensitivity of modern *h*-BN–based photodetectors is below 240 nm due to insufficient photon energy for the excitation of localized electrons and their transition to the conduction band [263,264,265]. The detection bandwidth of *h*-BN photodetectors in the UV range can be extended by the surface plasmon resonance (SPR) effect. The use of Al as NPs in the *h*-BN/Al composite made it possible to extend the sensitivity to UV radiation to a wavelength of 266 nm [266]. Due to a localized SPR effect, Al NPs-supported BNNSs showed a significant increase in the illumination current in the deep UV region at a wavelength of 210 nm and a 60% improvement in sensitivity at a low input voltage [267].

## 7. Nanoelectronic, Tunnel, and Memory Devices

One-dimensional *h*-BN is a promising material for various nanoelectronic devices. This is due to the ability to change the material conductivity by adding interstitial or substitutional defects at its edges. As a result, metallic, half-metallic, and semiconducting properties of *h*-BN were reported [268,269]. A crystalline 2D insulator of the *h*-BN type with a bandgap of ~5.95 eV is often used as a substrate for Gr in field-effect transistors [270]. Ultrathin *h*-BN (≤6 layers) is used in tunnel devices [271,272]. Defect-induced electron transport in the O-doped edge of *h*-BN was recently reported [273]. Compared to the pristine *h*-BN, the O-doped edge shows an increase in current by two orders of magnitude. BN is often added to various electronic devices as an insulating layer. For example, the Gr/*h*-BN/n-Si heterostructure was characterized as a planar electron emission device with a high electron beam monochromaticity and high electron emission current density, in which, the *h*-BN layer effectively suppressed the inelastic scattering of electrons [274].

Dielectrics with low losses at microwave frequencies are essential for highly coherent solid-state quantum computing platforms. Hexagonal BN was utilized as a low-loss dielectric for superconducting quantum circuits and qubits [275]. NbSe_2_–*h*-BN–NbSe heterostructures were placed in plane-parallel capacitors integrated into superconducting circuits. The integration of plane-parallel capacitors with aluminum Josephson junctions made it possible to obtain transmon qubits with a coherence time of 25 μs.

The utilization of *h*-BN significantly improved the performance of 2D n-type SnS/*h*-BN field-effect transistors [276]. The authors applied a surface oxide conversion method using highly reactive Ti to prevent SnS oxidation. As a result, an extremely high field effect mobility of 87.4 cm^2^V^−1^s^−1^ was achieved. BN encapsulation is a promising approach to preserve the ultra-clean interface and intrinsic charge transport in semiconductors. Such BN-encapsulated field-effect transistors with unipolar MoS_2_ and ambipolar WSe_2_ channels demonstrated a high overall electronic performance (low density of interfacial charged impurities ~10^11^ cm^−2^ and high charge mobility > 1000 cm^2^ V^−1^ s^−1^ at low temperatures) [277]. Although dry transferred *h*-BN is widely used as an encapsulation layer for various electronic devices, a lack of interfacial flatness can degrade the contact performance. *h*-BN can significantly affect the interface structure, causing deformation and strains [278]. Thinner *h*-BN can fit closer to metal contacts, creating smaller deformation areas.

Hexagonal BN found application in memristor devices that perform artificial synaptic functions [279]. Obtained Ag/*h*-BN/Ag planar synaptic devices exhibited a low operating current of 100 pA and low energy consumption per synaptic event of ~170 fJ per pulse. In addition, the device efficiently mimicked the short-term and long-term plasticity behavior required for neuromorphological systems. A flexible low-dimensional *h*-BN-based memristor has been developed, which has a non-volatile memory with ultra-low power consumption, capable of simultaneously performing digital mem- and neuromorphic calculations [280]. The memristor power consumption per synaptic event was low (fJ level), and the response time (1 ms) of neuromorphic computations was four orders of magnitude shorter than that of the human brain (10 ms).

Ultrafast operation (20–21 ns) in flash memory devices containing *h*-BN has been demonstrated [281,282]. Although it is believed that ultrafast operation requires an atomically smooth interface [281], a recent study suggests that the high breakdown dielectric strength of *h*-BN allows for operation at a higher tunneling current and the ability to achieve ultrafast operations [283].

## 8. Energy Materials and Batteries

BNNSs were utilized as an insulating layer preventing electrical contact between the anode and cathodes in a lithium-ion battery [284]. The resulting composite membranes showed a high ionic conductivity and cycling stability, which make it possible to use batteries at an extremely high temperature of 120 °C. Dielectric polymer nanocomposites with a high energy efficiency and energy density were obtained by combining a low-loss polymer matrix (poly(vinylidene fluoride−hexafluoropropylene) and poly(methyl methacrylate)) and lysozyme-modified BNNSs. The increased energy storage capacity is associated with an improved compatibility due to the strong particle/matrix interface and barrier effects preventing charge redistribution [285]. An *h*-BN dielectric coating on a lithium anode has been used as an interfacial layer to improve the stability and extend the life of a Li metal battery by suppressing the dendrite growth during the electrochemical plating/stripping process [286].

Bilayer *h*-BN/polymer capacitors with a high energy density and low loss were obtained by self-assembly at the oil–water interface [287]. The BN layer promotes energy storage by reducing dielectric loss and preventing charge transport, and extends the operating temperature ranges. Hexagonal BN has been characterized as a promising material for sulfur cathodes in lithium–sulfur batteries [288,289]. Nitrogen vacancies in few-layer BN developed as a cathode matrix for Li-S batteries promoted the immobilization and conversion of Li-S and accelerated the diffusion of Li ions [288]. rGO microspheres decorated with *h*-BNNSs showed a high initial capacity (1137 mAh g^−1^ at 0.2 C) and improved cycling stability (572 mAh g^−1^ at 1 C after 500 cycles) [289]. BN@N-CN sandwich structures, in which, BNNPs are evenly wrapped with N-doped CN NSs, demonstrated a high capacity of 574 mA × h × g^−1^ after 400 cycles at a current of 0.5 A g^−1^, with a Coulombic efficiency of 98% [290]. It is assumed that the strong BN chemisorption promotes the capture of polysulfides in the cyclic process and the acceleration of redox reactions.

## 9. Additive to Liquid Lubricants

Pure and surface-modified *h*-BN nanomaterials are promising green oil additives. Reduced friction and wear is achieved by continuously delivering BN nanostructures to the contact zone and forming a BN-based tribofilm. Mechanisms for reducing friction and wear in an oil suspension depend on the NPs size, shape, and concentration, as well as on their mechanical strength, thermal conductivity, and chemical stability. Hexagonal BNNPs can provide self-lubricating characteristics either due to their layered structure or through additional lubrication mechanisms, such as rolling, sliding, and exfoliation. To improve the dispersibility of NPs in lubricating oil, the *h*-BNNSs were subjected to alkali-assisted hydrothermal exfoliation followed by grafting with cetyltrimethylammonium (CTA) bromide [291]. The good dispersibility was attributed to the van der Waals interaction between the hydrocarbon in the oil and the hexadecyl chain/methyl groups of CTA. As an oil additive, ball-milled and alkyl-functionalized BNNSs showed a 45% reduction in friction and an 89% reduction in wear [292].

**Figure 6:**
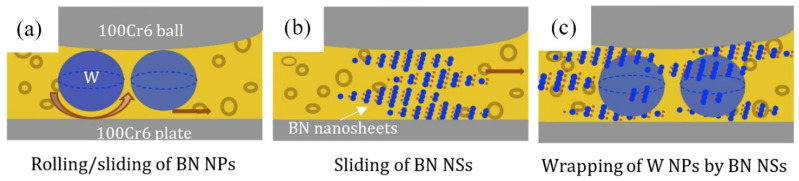
Schematics of friction mechanisms when adding (**a**) W NPs, (**b**) BN NSs, and (**c**) W NPs and BN NSs to PAO6 oil. Adapted with permission from Ref. [294]. Copyright 2020, Elsevier.

As additives (0.01 and 0.1 wt%) to PAO6 oil, BNNPs of various morphologies (hollow particles with a smooth surface, solid particles with a petal surface, and globular particles formed by numerous thin *h*-BNNSs) were studied [293]. In situ TEM compression tests have shown that, under an applied load, globular NPs disintegrate into individual nanosheets, lining up parallel to the friction direction, thereby minimizing the shear force. In situ TEM mechanical tests of hollow particles with a smooth surface have shown that they withstand a high contact pressure of 1.0–1.5 GPa and provide lubrication through rolling/slip mechanisms. The simultaneous use of several types of NPs makes it possible to further improve the tribological characteristics of oil-based lubricants. For example, the synergistic effect from the simultaneous addition of W NPs and BNNPs was associated with the rolling and sliding of W NPs and exfoliation and sliding of *h*-BN layers (Figure 6) [294]. In addition, the formation of core/shell W@BN structures by wrapping W NPs in *h*-BN sheets provides excellent lubricity at the macro level.

In addition to oils, the behavior of BNNPs in a humid environment and in water was studied. The obtained results showed that hydroxylation and polydopamine modifications help to reduce the friction coefficient and the wear rate of *h*-BNNPs as water additives [295]. The reduced friction of *h*-BN coatings in a humid environment was attributed to the dissociative adsorption of molecules containing −OH functional groups at *h*-BN defects [296].

## 10. Aerogels and Iongels

Another promising area of *h*-BN application is the creation of aerogels that are attractive for thermal insulation but suffer from poor mechanical properties. Aerogels with a high porosity and low density are usually obtained by molecular or assembly methods from molecular precursors and ready-made nanocrystals, respectively. The assembly scheme was utilized to prepare superhydrophilic and superhydrophobic BN aerogels consisting of various superstructures (nanoribbons and nanofibers) [297]. The ionogel based on exfoliated *h*-BN nanoplatelets showed a high ionic conductivity and mechanical strength [298]. Using the *h*-BN ionogel as a dielectric, fully printed thin-film transistors were manufactured [299].

The *h*-BN aerogel, obtained from a Gr aerogel template, had an ultralow density (~0.1 mg/cm^3^), high thermal stability (900 °C in air and 1400 °C in vacuum), superelasticity (up to 95%), and thermal superinsulation (~2.4 mW/m × K in vacuum and ~20 mW/m × K in air) [300]. The infiltration of various polymers into *h*-BN aerogels can impart new functional properties to the material. For example, the thermal conductivity (from insulator to conductor) and mechanical properties of *h*-BN/polymer composite aerogels can be controlled by the infiltration of various additives [301]. A high porous aerogel film consisting of *h*-BN nanoribbons intertwined and interconnected to each other, forming a 3D open-cell porous network, was obtained by the successively assembly of a molecular precursor, sublimation drying, and pyrolysis reaction [302]. This *h*-BN film has been characterized as a promising heat-insulating protective layer for portable electronic devices, which can prevent heat transfer to the skin, reducing the discomfort caused by heat.

## 11. Theoretical Insights

**Figure 7:**
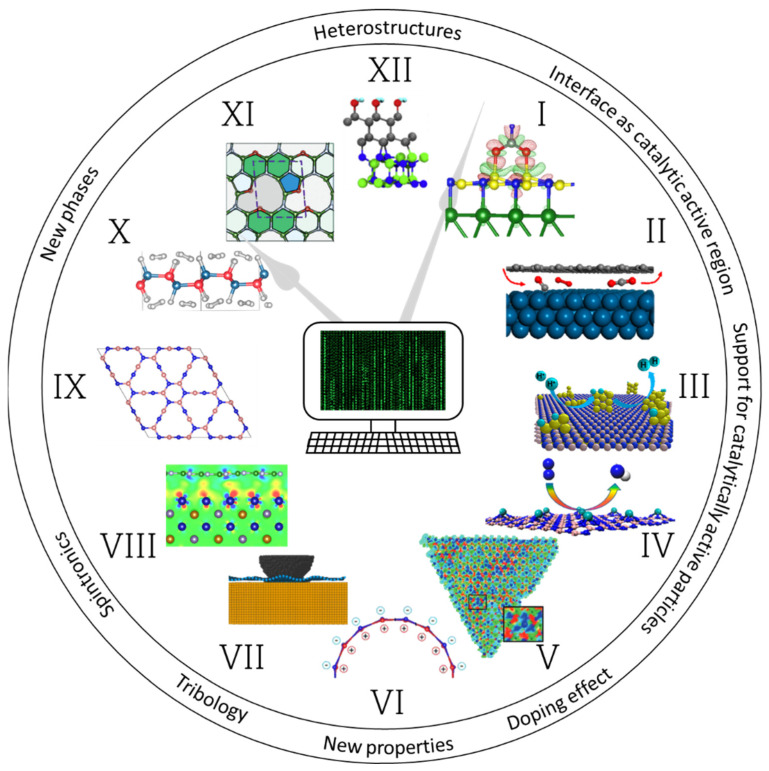
Schematics of various directions of modeling BN nanostructures (I, II—*h*-BN/metal interface as a catalytic center; III, IV—*h*-BN substrate for catalytically active NPs; V—*h*-BN doping; VI–search for new *h*-BN properties; VII—*h*-BN in tribology; VIII—*h*-BN in spintronics; IX–XI—search for new *h*-BN materials; XII—BN-based heterostructures). (I) Charge density redistribution during HCOO adsorption on *h*-BN/Ni(111). The image reprinted with permission from Ref. [303]. Copyright 2018, American Chemical Society. (II) Surface reaction at the *h*-BN/Pt(111) interface. The image reprinted with permission from Ref. [305]. Copyright 2015, American Chemical Society. (III) Catalytic activity of Au(111) clusters supported by *h*-BN in HER. The image reprinted with permission from Ref. [307]. Copyright 2021, American Chemical Society. (IV) Transition metal SACs in *h*-BN for ammonia production. The image reprinted with permission from Ref. [309]. Copyright 2017, American Chemical Society. (V) Negative charge density distribution around the O impurity in *h*-BN. The image reprinted with permission from Ref. [214]. Copyright 2017, WILEY-VCH Verlag GmbH & Co. KGaA, Weinheim. (VI) Electric polarization in *h*-BN during deformation (flexoelectricity). Adapted with permission from Ref. [322]. Copyright 2022, Elsevier. (VII) Friction in *h*-BN/Cu(001). Adapted with permission from Ref. [323]. Copyright 2022, Elsevier. (VIII) Interfacial charge redistribution at the *h*-BN/semimetal interface. Adapted with permission from Ref. [326]. Copyright 2022, The Royal Society of Chemistry. (IX) BN as an analog of graphyne. Adapted with permission from Ref. [327]. Copyright 2018, Elsevier. (X) Hydrogenated BN. The image reprinted with permission from Ref. [331]. Copyright 2021, American Chemical Society. (XI) B_5_N_3_O. Adapted with permission from Ref. [335]. Copyright 2021, The Royal Society of Chemistry. (XII) Cr/*h*-BN heterostructure. Adapted with permission from Ref. [340]. Copyright 2019, Elsevier.

In the field of catalysis, *h*-BN is usually studied as a substrate for catalytically active NPs. However, the metal/*h*-BN interface can also be considered as a catalytic site. It is theoretically predicted that *h*-BN/Ni(111) and *h*-BN/Co(0001) should have very good electrocatalytic activities in the reduction of CO_2_ to HCOOH (Figure 7-I [303]). An *h*-BN/TiO_2_(100) heterostructure was considered in the DFT calculations as a promising photocatalyst for water splitting [304]. It was shown that the transition of photogenerated electrons from *h*-BN to TiO_2_ causes the formation of a built-in electric field and band bending, which leads to a recombination of electron–hole pairs at the interface and charge accumulation in the conduction band of *h*-BN and the valence band of TiO_2_. Hexagonal BN shells can interfere with chemical reactions. For example, in the *h*-BN/Pt(111) system, the interaction of CO with Pt is weakened due to the shielding effect of *h*-BN, which reduces the CO poisoning of the metal, leading to increased CO oxidation at the *h*-BN/Pt(111) interface (Figure 7-II [305]).

Although the size of catalytically active NPs significantly exceeds the size of an atom (in the limiting case, this is SACs), the high chemical activity and undercoordination of metal atoms contribute to the activation of a chemical reaction. In simulations, the size of a metal cluster is reduced to a few atoms due to natural computational power limitations. It was proposed to deposit Pt_4_ on the BN surface for CO oxidation [91], Ni_2_ for dry methane reforming (DMR) [306], and Au_1–8_ for _the_ hydrogen evolution reaction (HER) (Figure 7-III) [307]. The general inertness of *h*-BN creates the problem of anchoring the deposited metal clusters. Defects are usually used in theoretical calculations and, most often, the simplest monovacancy defect is considered [93,306], although, in real conditions, the topology of *h*-BN is much more complicated. It has been proposed to use the *h*-BN grain boundary as an adsorption site for SACs construction [308].

A single atom is the most promising (and convenient for simulation) catalyst. Its free energy is maximal due to unsaturated bonds, quantum confinement effects, and the sparse quantum levels, resulting in unique chemical properties. Systematic DFT studies of single transition metal atoms (Sc to Zn, Mo, Ru, Rh, Pd, and Ag) supported on an *h*-BN monolayer with a boron monovacancy were performed in order to assess the possibility of obtaining ammonia (NH_3_) from molecular dinitrogen (Figure 7-IV) [309]. The Ag atom can be anchored to a boron defect, which makes CO oxidation thermodynamically favorable in a wide temperature range [310]. BN containing Pt, Ag_1_Pt_1_, and Ag_2_Pt_2_ exhibits high activity in HER [98]. Coordinated B bonded to Mo at the *h*-BN grain boundary greatly alters the spin states and bond strengths within some intermediates, resulting in superior activity in the nitrogen reduction reaction (NRR) [308].

It is important to note that it is not only a metal atom that can act as a catalyst. For example, a Si atom embedded in *h*-BN effectively captures CO_2_ [311]. The *h*-BN edge can also be considered as a catalytic site due to its natural chemical activity. Two mechanisms have been proposed for the hydrolysis of the *h*-BN edge [312]. Doping an edge with carbon can further increase the chemical activity, as was shown in the Knoevenagel condensation of benzaldehyde with malononitrile to produce benzylidene malononitrile [87]. It is assumed that the reaction proceeds according to the dissociative adsorption mechanism on the oxygen-terminated BCN edge, in which, the C-H bond is broken, and the proton and the rest are adsorbed on neighboring O sites bonded with edge carbon. This promotes the desorption of the intermediates in order to form the final product.

A vacancy defect is often considered an active site. Simulations showed that *h*-BN/Cu(111) with a boron vacancy can be a potential catalyst for the oxygen reduction reaction (ORR) [313]. A favorable CO oxidation reaction on *h*-BN/Ni(111) and *h*-BN/Cu(111) with nitrogen and boron vacancies, respectively, was predicted. The photocatalytic degradation of CF_3_COOH on *h*-BN was explained by the presence of boron and nitrogen vacancies acting as strong Lewis acid and base sites, respectively, which activate the *h*-BN plane for the photocatalytic dissociation of the C−F bond [314]. Although these vacancies are likely to be deactivated during the photocatalytic reaction, oxygen-filled nitrogen vacancies reduce the strength of the active sites and contribute to CF_3_COOH decomposition. Molecular interactions and adsorption mechanisms of steroidal estrogens (estrone, E1; 17β-estradiol, E2 and estriol, E3) and anti-estrogens (bisphenol-A, BPA) on pure and defective *h*-BN surfaces were recently analyzed [315]. Steroid contaminants chemisorb to nitrogen vacancy in *h*-BN due to short bond distances and high adsorption energies. The concept of dangling bonds was also used to explain the experimentally observed magnetism in *h*-BN [213]. A sharp increase in the total magnetic moments upon the introduction of nitrogen vacancies was theoretically predicted. Ab initio modeling also explains the SFEs in boron vacancy-derived defect centers of *h*-BN [316]. Note that the short lifetime of vacancies hardly makes it possible to consider them as a promising system for the implementation of any physicochemical process.

Doping is one of most promising approaches for tuning *h*-BN properties. It has been predicted that doping with transition metals (Ti, Cr, Fe, Mo, and W) changes the electronic properties of *h*-BN and increases the PH_3_ detection capacity [317]. It was shown that an in-plane *h*-BN/Gr nanoarray can selectively adsorb and, therefore, separate four types of single nucleotides [318] and gas molecules (NH_3_ and NO_2_) [319]. Sr-, Ba-, and Ca-doped *h*-BN monolayers were found to be energetically stable and become superconductors with T_c_ values of 5.83 K, 1.53 K, and 12.8 K, respectively, which are much higher than those of Ca-, Sr-, and Ba-doped Gr [320]. The observed paramagnetic properties in BNO NSs are explained by localized negative charges around the O impurities, which are caused by the injection of additional electrons into the conjugated π bonds from the O atoms with respect to the removed N atoms [214] (Figure 7-V). Doping with carbon increased the magnetic moment of *h*-BN by a factor of approximately two [217]. This is due to the uniform distribution of Gr QDs and the formation of C-N and C-B bonds, which leads to spin polarization and charge asymmetry.

Interesting effects are also observed in undoped *h*-BN multilayers, which can bend without breaking, creating a large deformation gradient. By analogy with Gr, the deformation gradient leads to electric polarization, i.e., the appearance of the flexoelectric effect (Figure 7-VI). A linear dependence of flexoelectric dipole moments on the local curvature in various *h*-BN nanostructures (nanotubes and fullerenes) was theoretically established, and the value of the flexoelectric coefficient, close to the corresponding value in Gr, was obtained [321]. Warping caused by bending can significantly enhance the flexoelectric effect by introducing a negatively charged outer N-wall and a coaxial positively charged inner B-wall [322].

The tribological properties of *h*-BN-coated Cu were studied using the molecular dynamic simulation (MDS). It is shown that the friction coefficient of the Cu(001) surface coated with *h*-BN decreases, whereas the wear resistance increases. The presence of *h*-BN reduces the interaction of Cu with the counterpart material, the van der Waals interfacial force, the Von Mises stress, and the temperature in the tribocontact zone (Figure 7-VII) [323]. Using large-scale MDS based on machine learning potentials, the hydrodynamic behavior of water in BNNTs and CNTs was compared [324]. The sticky behavior of water on the BN surfaces was explained by their special chemical composition and polarity, which affect the hydrogen atoms.

Based on recent experimental results of the synthesis of the Gr/semimetal Co_2_Fe_1/2_Ge_1/2_Ga_1/2_ heterostructure [325], a theoretical study of its combination with an *h*-BN monolayer was carried out (Figure 7-VIII) [326]. The observed large spin polarization near the interface and strong magnetization confirm the promise of novel *h*-BN/Heusler alloy heterojunctions for spintronics.

Theoretical modeling is used to search for new BN-based structural configurations. This is facilitated by the similarity between carbon and BN, which suggests the presence of similar phases (not necessarily as stable), such as diamond and c-BN, lonsdaleite and w-BN, graphite and *h*-BN, and carbon (carbyne) and BN chains. Recently, a BN structure was theoretically proposed, similar to a graphyne, containing both sp^2^- and sp^1^-hybridized bonds (Figure 7-IX) [327]. An analogue of three-fold coordinated C networks (haeckelite) was also predicted for BN [328]. Unlike the carbon counterpart, whose structure consists of a combination of *sp*^2^-bonded carbon pentagons, hexagons, and heptagons, low energy structures in BN have tetragonal and octagonal defects.

Increased attention was paid to studying the structure of BN with homeopolar bonds. The presence of homeopolar bonds with tetragonal defects in a heckelite-like BN structure was predicted [329]. The material has four anisotropic Dirac cones in the first Brillouin zone exactly at the Fermi level. In addition, the obtained values of Young’s modulus and Poisson ratio indicate a high mechanical strength. Hexagonal BN was predicted, consisting of azo (N-N) and diboron (B-B) groups with a band gap of 2.446 eV at GW level, capable of producing hydrogen molecules in the photocatalytic water-splitting reaction (WSR) [330]. The increased mobility of electrons and holes in the zigzag direction of this material is much higher than that of many other 2D semiconductors. However, the presence of energy-unfavorable homogeneous bonds may indicate its instability.

Continuing the comparison with carbon, one would expect to obtain *h*-BN derivatives such as boron nitride hydride, fluoride, or oxide. However, a direct correspondence should not be expected due to the different interactions of polar BN bonds with functional groups. The formation of hydrogenated *h*-BN from ammonia borane is predicted only at high pressures above 36 GPa (Figure 7-X) [331]. Fluorinated *h*-BN was recently synthesized experimentally [210,332] and confirmed by theoretical calculations, taking into account various possible configurations of the fluorinated *h*-BN structure and their energy states [212]. In addition to changing the electronic properties, fluorine causes a significant redistribution of the charge density of nitrogen atoms, which leads to ferromagnetic ordering.

A number of works considered the mechanism of *h*-BN oxidation at temperatures above 600 K [333,334]. Oxygen is usually built into the *h*-BN lattice; however, at its maximum concentration (B_5_N_3_O), *h*-BN can change its structure: in addition to six-membered rings, one can expect pentagons with a homoatomic bond between nitrogen atoms (Figure 7-XI) [335].

New BN nanostructures can be obtained by vertical self-assembly of the layers. In this case, BN behaves in the same way as carbon, for which, a theory has been developed that predicts that the adsorption of hydrogen, fluorine, etc., atoms on the Gr surface facilitates the assembly process, and, for the limiting case of two layers, the process becomes barrier-free [336]. Experimental confirmation of the effect of a chemically induced phase transition in carbon [337] prompted the study of the closest analogue of carbon, BN, but only recently have experimental and theoretical studies appeared in which the effect of the *h*-BN → c-BN phase transformation was demonstrated [338,339]. MDS has shown that the application of local pressure can lead to the joining of *h*-BN layers [338]. The local compression of two *h*-BN layers in the presence of hydroxyl ions can transform the insulator into a conductive material [339].

The close lattice parameter values of *h*-BN and Gr make it possible to consider the possibility of obtaining Gr/*h*-BN heterostructures (Figure 7-XII) [340]. The passivation of the *h*-BN surface with hydroxyl groups plays a decisive role in facilitating the layer connection. The theory predicts that, by varying the *h*-BN and Gr layers, it is possible to tune the heterostructure band gap in a wide range from 0 to 5 eV [341].

## 12. Final Remarks

In conclusion, it can be noted that, although, over the past few years, significant progress has been made both in the field of synthesis and application and theoretical modelling of new types of 0D, 1D, 2D, and 3D *h*-BN nanomaterials and *h*-BN-based hybrid nanomaterials and heterostructures, some important challenges should still be resolved. So far, there are no large-scale methods for obtaining high-quality BN nanostructures with a certain aspect ratio. The solution of this problem will make a significant contribution to the production of composites based on metals, ceramics, and polymers with improved mechanical properties and thermal conductivity. The development of areas associated with the use of *h*-BN emulsions is still constrained by the tendency of NPs to agglomerate. Therefore, more reliable methods of *h*-BNNP surface functionalization are needed to obtain stable suspensions. The formation of hierarchical ceramic structures and three-dimensional ceramic frameworks in metal matrix composites is a promising direction in the development of materials with unique thermomechanical properties. This, in turn, requires a detailed in situ study of the reactions between the ceramic phases and the metal matrix, as well as the formation and growth of secondary phases. Many of the unique properties of *h*-BN are associated with the presence of defects and impurity atoms that form QDs, SACs, SPEs, etc. Therefore, the proper engineering of intrinsic defects and doping atoms in *h*-BN in order to tune the desired material properties for a particular application remains extremely urgent. Further progress in the creation of new efficient nanocatalysts is impossible without a deep understanding of the nature of active sites and the mechanisms of catalytic reactions. The nature of QEs in *h*-BN has not been fully elucidated, since the generation and activation mechanisms remain poorly understood. As a result, QDs and SPEs are often produced empirically, without proper theoretical insight. In addition, the localization of QEs in *h*-BN faces technological challenges.

## Figures and Tables

**Table 1:** Antibacterial activity of BN NPs and BN-based nanohybrids.

Material	BNNP Content (%)	Pathogens	Ref.
PNMPy-BNNPs	10.0	*E. coli*, *S. aureus*, *P. aeruginosa*, *E. faecalis*	[124]
LDPE-BNNPs	5.0–20.0	*E. coli*, *S. aureus*, *P. aeruginosa*, *S. epidermidis*	[125]
PHA/CH-BNNPs	0.1–1.0	*E. coli* K1 Methicillin-resistant *S. aureus*	[126]
QAC-BNNPs-PP	3.0–10.0	*E. coli* Carolina #155065A *S. aureus* Carolina #155556	[127]
CEL-BNNPs	1.0–3.0	*E. coli* K12 (ATCC 29425) *S. epidermidis* ATCC 49461	[128]
	MIC of BN (mg/mL)		
BNNPs	15	Multidrug resistant *E. coli* (12 strains)	[129]
BNNPs	1.62	*S. mutans* 3.3	[130]
	400	*S. mutans* ATTC 25175	
	400	*S. pasteuri* M3	
	3.25	*Candida* sp. M25	
BNNPs	256	*E. coli*	[131]
	128	*B. cereus*	
	128	*S. aureus*	
	128	*E. hirae*	
	128	*P. aeruginosa*	
	256	*L. pneumophila* subsp. *pneumophiia*	
	256	*C. albicans*	
BNNSs	100	*E. coli* DH5α	[132]

Poly(N-methylpyrrole (PNMPy), polyhydroxyalkanoate (PHA), cellulose (CEL), chitosan (CH), polypropylene (PP), quaternary ammonium salt (QAC), low-density polypropylene (LDPE).

## Data Availability

Not applicable.

## Abbreviations

Al matrix composites	AlMCs
BE	Binding energy
BNCT	Boron neutron capture therapy
BNNPS	BN nanoparticles
BNNSs	BN nanosheets
BNNTs	BN nanotubes
CH	Chitosan
CNTs	Carbon nanotubes
CTA bromide	Cetyltrimethylammonium bromide
CVD	Chemical vapor deposition
DFT	Density functional theory
DMR	Dry methane reforming
DMF	Dimethylformamide
DMSO	Dimethyl sulfoxide
DOX	Doxorubicin
FA	Folic acid
Gr	Graphene
HER	Hydrogen evolution reaction
HDF cells	Human dermal fibroblast cells
HUVE cells	Human umbilical vein endothelial cells
IPA	Isopropyl alcohol
MB	Methylene Blue
MDS	Molecular dynamic simulation
MMCs	Metal matrix composites
NHPI	N-hydroxyphthalimide
NPs	Nanoparticles
NRR	Nitrogen reduction reaction
ORR	Oxygen reduction reaction
PBS	Phosphate-buffered saline
Pc	Phthalocyanine
PET	Polyethylene terephthalate
PLA	PLA
PVA	PVA
QD	QD
QEs	QEs
rGO	rGO
RhB	RhB
ROS	ROS
SACs	SACs
SMSI	SMSI
SPEs	SPEs
SPR	SPR
SPS	SPS
TA	TA
TOF	TOF
WSR	WSR
